# Zeolite Membrane‐Based Low‐Temperature Dehydrogenation of a Liquid Organic Hydrogen Carrier: A Key Step in the Development of a Hydrogen Economy

**DOI:** 10.1002/advs.202403128

**Published:** 2024-06-13

**Authors:** Sejin Kim, Seungmi Lee, Suhyeon Sung, Sangseo Gu, Jinseong Kim, Gihoon Lee, Jaesung Park, Alex C. K. Yip, Jungkyu Choi

**Affiliations:** ^1^ Chemical & Biological Engineering Korea University 145 Anam‐ro, Seongbuk‐gu Seoul 02841 Republic of Korea; ^2^ Green Carbon Research Center Korea Research Institute of Chemical Technology (KRICT) 141 Gajeong‐ro, Yuseong‐gu Daejeon 34114 Republic of Korea; ^3^ Chemical and Process Engineering University of Canterbury Christchurch 8140 New Zealand

**Keywords:** H_2_ separation, liquid organic hydrogen carrier (LOHC), membrane reactor, methylcyclohexane (MCH) dehydrogenation, zeolite membrane

## Abstract

Methylcyclohexane (MCH) dehydrogenation is an equilibrium‐limited reaction that requires high temperatures (>300 °C) for complete conversion. However, high‐temperature operation can degrade catalytic activity and produce unwanted side products. Thus, a hybrid zeolite membrane (Z) is prepared on the inner surface of a tubular support and used it as a wall in a membrane reactor (MR) configuration. Pt/C catalysts is packed diluted with quartz sand inside the Z‐coated tube and applied the MR for MCH dehydrogenation at low temperatures (190–250 °C). Z showed a remarkable H_2_‐permselectivity in the presence of both toluene and MCH, yielding separation factors over 350. The Z‐based MR achieved higher MCH conversion (75.3% ± 0.8% at 220 °C) than the conventional packed‐bed reactor (56.4% ± 0.3%) and the equilibrium state (53.2%), owing to the selective removal of H_2_ through Z. In summary, the hybrid zeolite MR enhances MCH dehydrogenation at low temperatures by overcoming thermodynamic limitations and improves the catalytic performance and product selectivity of the reaction.

## Graphical abstract

A membrane reactor (MR) configuration is a unique solution for overcoming the equilibrium limitation of dehydrogenation of liquid organic hydrogen carriers. A robust H_2_‐permselective zeolite membrane is adopted as the wall in an MR, resulting in high methylcyclohexane conversion at low temperatures. According to the Le Chatelier's principle, this is attributable to selective H_2_ removal through the membrane during reaction.

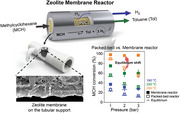

## 1 Introduction

H_2_ is a promising energy carrier for resolving environmental issues including climate change and air pollution.^[^ ^1^ ^]^ H_2_ can be produced by various methods, such as electrolysis, steam methane reforming, and biomass gasification.^[^ ^2^ ^]^ Notably, H_2_ is a clean energy source, producing only water when used in fuel cells or combustion engines.^[^ ^3^ ^]^ However, the storage and transport of H_2_ is economically challenging because it requires high pressures or low temperatures for compression.^[^ ^4^ ^]^ Liquid organic hydrogen carriers (LOHCs) are chemicals that can store and transport H_2_ in liquid form via the reversible hydrogenation and dehydrogenation of organic compounds, which are liquid at standard temperature and pressure.^[^ ^5^ ^]^ Thus, LOHCs can be stored under mild conditions, and, when H_2_ is needed, can be dehydrogenated to release H_2_, yielding H_2_‐lean LOHCs.^[^ ^6^ ^]^ The released H_2_ is used as an energy source, and the H_2_‐lean LOHCs can be hydrogenated again for further use. This enables a closed carbon cycle that minimizes energy waste.^[^ ^7^ ^]^

Methylcyclohexane (MCH) is an attractive LOHC because of its high H_2_ content (6.16 wt.%),^[^ ^8^ ^]^ low toxicity, and relatively low boiling point (101 °C), compared to other LOHCs, such as cyclohexane and decalin).^[^ ^9^ ^]^ However, the dehydrogenation of MCH is endothermic, requiring high temperatures (>300 °C) for complete conversion. Unfortunately, high temperatures can degrade the catalytic activity^[^ ^10^ ^]^ and decrease the target molecule selectivity by producing unwanted side products.^[^ ^7^, ^11^ ^]^ Therefore, the development of appropriate catalysts to solve these problems has drawn much attention.^[^ ^9^, ^10^, ^12^ ^]^ As an alternative solution, the use of a membrane reactor (MR) that combines reaction and separation in one device has been proposed to improve the reaction activity and, further, the coupling of two reactions.^[^ ^13^ ^]^ Crucially, MRs can shift the equilibrium state to the product side because a product is selectively separated from the reaction mixture, thus overcoming thermodynamic limits and achieving higher conversions at lower temperatures.^[^ ^1^, ^14^ ^]^ Further, the use of the MR configuration can increase MCH conversion and purify H_2_ molecules on the permeate side at low temperatures, reducing production and separation costs.^[^ ^15^ ^]^ Indeed, several examples of MCH dehydrogenation using MRs have been reported.^[^ ^9^, ^16^ ^]^

Robust inorganic materials that can withstand harsh chemical and high‐temperature conditions are good candidates for the H_2_‐selective membrane material in MRs, for example, metals such as palladium and its alloys^[^ ^17^ ^]^ and porous materials such as metal–organic frameworks,^[^ ^18^ ^]^ zeolites,^[^ ^19^ ^]^ carbon sieves,^[^ ^20^ ^]^ and silica.^[^ ^21^ ^]^ In particular, zeolites are promising MR wall materials owing to their well‐defined porous structures, high thermal and chemical stabilities, and molecular sieving ability.^[^ ^22^ ^]^ As an example, the deca‐dodecasil 3 rhombohedral (DDR) type zeolite has pore sizes of 0.36 × 0.44 nm^2^,^[^ ^23^ ^]^ which makes it attractive for the separation of H_2_ (kinetic diameter: 0.29 nm)^[^ ^24^ ^]^ from the larger MCH (0.60 nm^[^ ^25^ ^]^) and toluene (Tol; 0.59 nm^[^ ^25^ ^]^), which are present in the MCH dehydrogenation reaction. Crucially, continuous DDR zeolite membranes can be grown from a chabazite (CHA) type zeolite seed layer.^[^ ^26^ ^]^ However, although zeolite membranes have great potential for MRs, there has been little research in this applied area compared to that on conventional zeolite membranes for separation.^[^ ^13^, ^27^ ^]^ Specifically, to the best of our knowledge, to date, there have been no reports of the use of zeolite membranes in the MR configuration for MCH dehydrogenation.

In this study, we explored the use of a H_2_‐selective zeolite membrane as a wall in an MR for MCH dehydrogenation. Briefly, we grew a DDR zeolite film (referred to as DDR@CHA hybrid) from a CHA type zeolite seed layer deposited on the inner surface of an α‐alumina tubular support. The hetero‐epitaxial growth method based on the structural compatibility of two types of zeolites was key to forming a continuous DDR@CHA hybrid zeolite membrane.^[^ ^26a^ ^]^ Further, to ensure a fair comparison, we also used all‐glazed tubes, which are impermeable to all involved molecules, to evaluate the performance of a conventional packed‐bed reactor. The separation performance of the prepared DDR@CHA hybrid zeolite membranes was investigated using binary mixtures of H_2_/Tol and H_2_/MCH. The effects of MR‐relevant operating conditions (weight hourly space velocity, reaction temperature, and total pressure on the feed and permeate sides) on the performance and long‐term stability of the MR were also investigated. Finally, the performance of the zeolite‐based MR was compared with those of a packed‐bed reactor and the equilibrium state, as well as those of MRs reported in the literature.

## 2 Results and Discussion

### 2.1 Membrane Properties of a DDR@CHA Hybrid Zeolite Membrane (Z)

**Figure 1:**
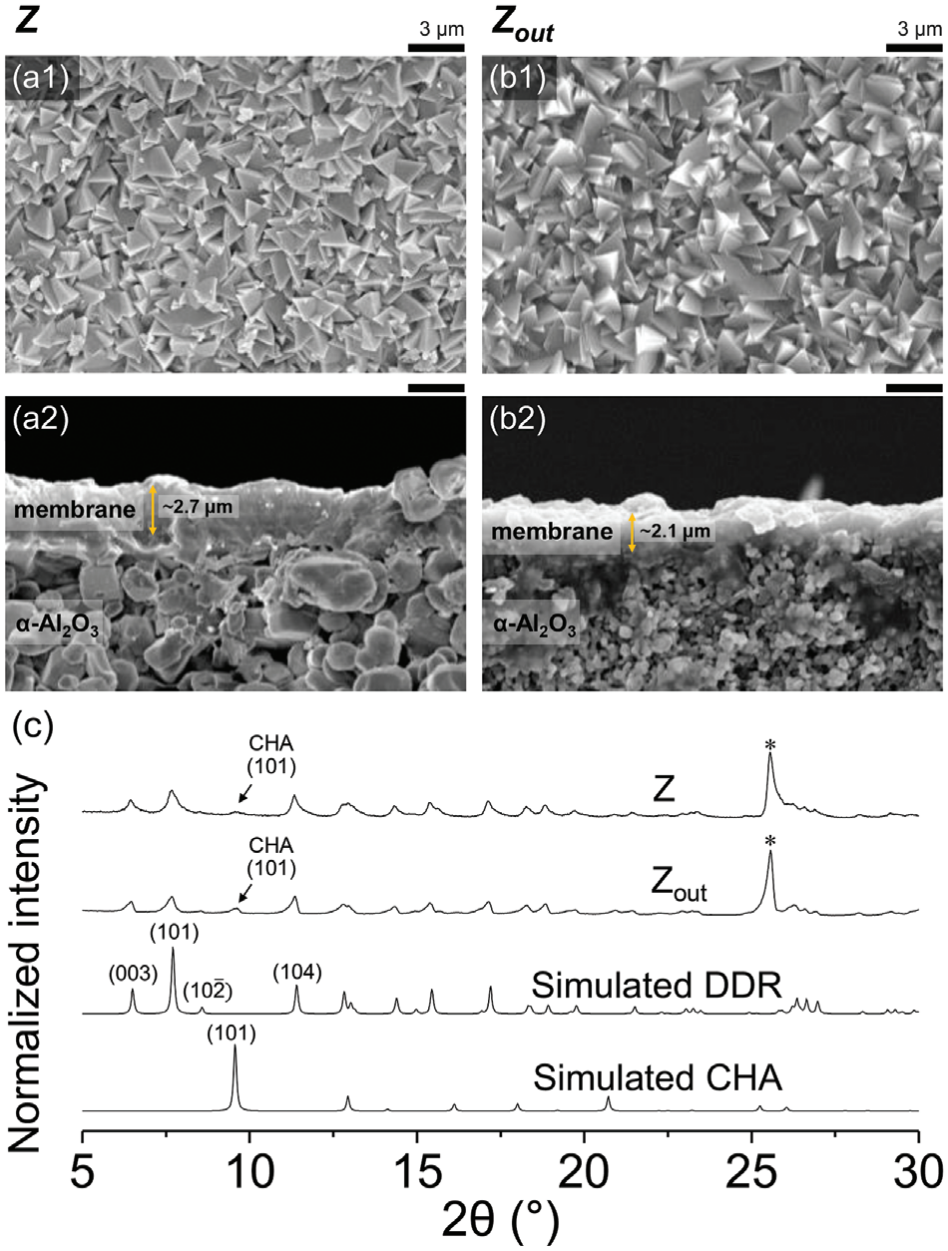
(a1)‐(b1) Top and (a2)‐(b2) cross‐sectional view SEM images of the DDR@CHA hybrid membranes on the (a1)‐(a2) inner (i.e., Z) and (b1)‐(b2) outer (i.e., Z_out_) surfaces of the α‐alumina tubular support. (c) XRD patterns of Z and Z_out_ along with the simulated XRD patterns of all‐silica DDR and CHA zeolites. In (a1)‐(b1) and (a2)‐(b2), scale bars above the SEM images indicate 3 µm. Thicknesses of zeolite membranes in (a2) and (b2) are denoted by yellow arrows. In (c), the XRD peaks corresponding to the (101) plane in the CHA zeolite, owing to its minor co‐presence in Z and Z_out_, are indicated by arrows and the asterisk (^*^) indicates the XRD peak arising from the α‐alumina tubular support.

DDR zeolite membranes heteroepitaxially grown from the CHA seed layers were prepared on the inner and outer surfaces of α‐alumina tubular supports, denoted Z and Z_out_, respectively. Z_out_ is identical to that of the DDR@CHA hybrid zeolite membrane reported by us previously.^[^ ^26b^ ^]^ Detailed information regarding the zeolite membranes and their use for MRs is given in Subsection S1 (Supporting Information). The scanning electron microscopy (SEM) images in **Figure** **1**a,b shows that both Z and Z_out_ were continuous on the tubular supports, and the pyramidal spike‐like grains were comparable to those of pure DDR membranes.^[^ ^28^ ^]^ Z and Z_out_ had similar membrane thicknesses, ≈2.7–2.1 µm, respectively, suggesting that they may show comparable separation performance.^[^ ^26a^ ^]^ The X‐ray diffractometry (XRD) patterns in Figure 1c confirm that Z and Z_out_ contained a large amount of DDR zeolite and some CHA zeolite. For clarity, the (101) reflection of CHA zeolite is indicated by an arrow in Figure 1c. These results confirm the synthesis of the DDR@CHA hybrid zeolite membrane^[^ ^26b^ ^]^; indeed, the presence of a minor CHA zeolite component was a result of the CHA seed layer (≈0.2–1 µm^[^ ^26b^ ^]^) remaining in the final hybrid zeolite membrane (≈2–3 µm as shown in Figure 1b1‐b2). The SEM and XRD analyses confirm that intact zeolite membranes comprising mostly DDR‐type zeolite were formed on the inner and outer surfaces of the α‐alumina tubular supports.

**Figure 2:**
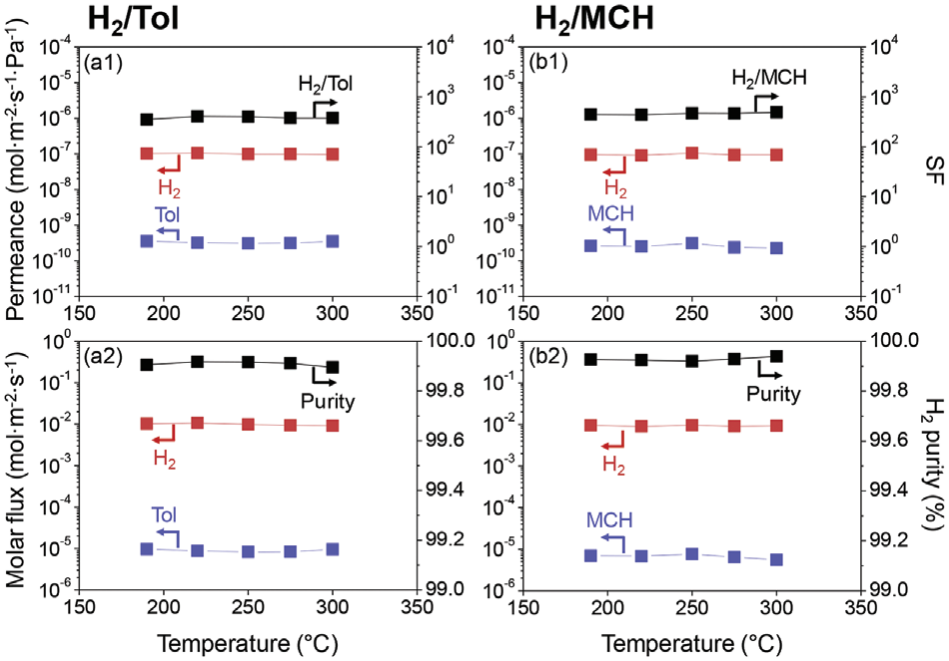
(a1)‐(b1) Permeances and SFs through Z and (a2)‐(b2) the corresponding molar fluxes and H_2_ purities measured at (a1)‐(a2) H_2_/Tol and (b1)‐(b2) H_2_/MCH molar ratios of 3:1 in the mixed feed. Separation performance tests were performed with 50 mL min^−1^ of Ar sweep gas at different temperatures (i.e., 190, 220, 250, 275, and 300 °C) at 1 bar on the feed side. For the H_2_ purity in (a2)‐(b2), the Ar sweep gas was excluded from the calculation. Data shown here are averaged results of three independent samples and error bars (representing the corresponding standard deviations) are included for all data points. For clarity, the error bars that are embedded in the symbols (so not conspicuous) are owing to the low standard deviation values.

### 2.2 Separation Performance of Z

The membrane properties of Z and Z_out_ were comparable to those of the DDR@CHA membrane reported previously.^[^ ^26b^ ^]^ Nevertheless, we tested the CO_2_/CH_4_ separation performance, which represents the quality of 8‐membered‐ring zeolite membranes at the bulk scale.^[^ ^29^ ^]^ As shown in Figure S1 (Supporting Information), both Z and Z_out_ had excellent membrane qualities, having CO_2_/CH_4_ separation factors (SFs) at 30 °C as high as ≈529 ± 45 and 488 ± 110, respectively. As expected, these were comparable to those of the previously reported DDR@CHA membrane^[^ ^26b^ ^]^ (see Subsection S2.1, Supporting Information for details). In addition, we assessed the capability of Z to separate H_2_ from the reactants and products of the MCH dehydrogenation reaction (**Figure** **2**a1‐a2,b1‐b2; for clarity, the real values of permeances and separation factors of H_2_/Tol and H_2_/MCH through Z are given in Tables S1 and S2, Supporting Information, respectively. For a better understanding, the corresponding H_2_ molar fluxes and purities are summarized in Tables S1 and S2, Supporting Information as well). The H_2_‐permselectivity was marked: H_2_/Tol SFs of ≈380 from 190 to 300 °C suggested that Z is a promising high‐performance MR material (Figure 2a1).^[^ ^30^ ^]^ The high H_2_‐permselectivity resulted in H_2_ purities as high as ≈99.91% on the permeate side (Figure 2a2). Furthermore, the separation performance of Z for H_2_/MCH mixtures (because MCH is also present in the reaction) was comparable to that for the H_2_/Tol mixture. Specifically, the H_2_/MCH SFs were ≈460 (Figure 2b1), resulting in a H_2_ purity on the permeate side of ≈99.93% (Figure 2b2) at 190, 220, 250, 275, and 300 °C. The difference between the H_2_/MCH and H_2_/Tol SFs (i.e., slightly higher H_2_/MCH separation performance) could be attributed to the minute difference in the molecular size of MCH and Tol, ≈0.60 and 0.59 nm, respectively.^[^ ^25^ ^]^ Therefore, the H_2_/Tol separation performance of Z can be used as a metric to assess the separation of the main components (MCH, H_2_, and Tol) in the MCH dehydrogenation reaction.^[^ ^16j^ ^]^

H_2_/Tol separation was also carried out at different pressures (1–3 bar) to account for the increased pressure in the reactor (feed side). The higher pressure reduced both H_2_/Tol SFs from 346 ± 15 to 137 ± 33 (Figure S2a, Supporting Information) and H_2_ purities on the permeate side from 99.90% ± 0.003% to 99.78% ± 0.03% (Figure S2b, Supporting Information). This is possible because Tol might preferentially permeate through the few non‐zeolitic regions on the membrane at high pressures, despite the low defect density in Z, as evidenced by the high CO_2_‐permselectivities (Figure S1, Supporting Information). However, the increased H_2_ molar flux should be proportional to the increase in total pressure, as shown in Figure S2b (Supporting Information), which would facilitate H_2_ removal from the product stream in MR configuration and effectively shift the equilibrium state to the product side.

### 2.3 Z‐Based MR for MCH Dehydrogenation

Before demonstrating the proof‐of‐concept of the Z‐based MR to overcome the thermodynamic limitations of the MCH dehydrogenation reaction, we confirmed the equilibrium MCH conversions by performing the reaction in a quartz tube reactor (Q) as a reference. Specifically, at a weight hourly space velocity (WHSV) of 43 mg g^−1^ min^−1^, the MCH conversion in Q matched the calculated equilibrium values at 170–288 °C (Figure S3, Supporting Information). To investigate the potential for the enhanced mass transfer of produced H_2_ to the permeate side, we used the MR configuration with Z and Z_out_ and found that they showed comparable MCH conversions at 190, 220, and 250 °C (Figure S4, Supporting Information). However, there was a slight improvement in the MCH conversion (pronounced at low temperatures; see Figure S4, Supporting Information), which could be ascribable to the facile permeation of H_2_ from the catalyst bed to the zeolite membrane on the inner surface of the tubular support.^[^ ^31^ ^]^ The use of the same zeolite membranes but in different positions will allow for differentiating the transport rate of the fast permeation species (here, H_2_), which is critical for determining the MR performance^[^ ^15^, ^32^ ^]^ (Figure S5 (Supporting Information); for clarity, the real values of permeances and separation factors of H_2_/Tol and H_2_/MCH through Z and Z_out_ are given in Tables S3 and S4, Supporting Information, respectively. For a better understanding, the corresponding H_2_ molar fluxes and purities are also summarized in Tables S3 and S4, Supporting Information).

**Figure 3:**
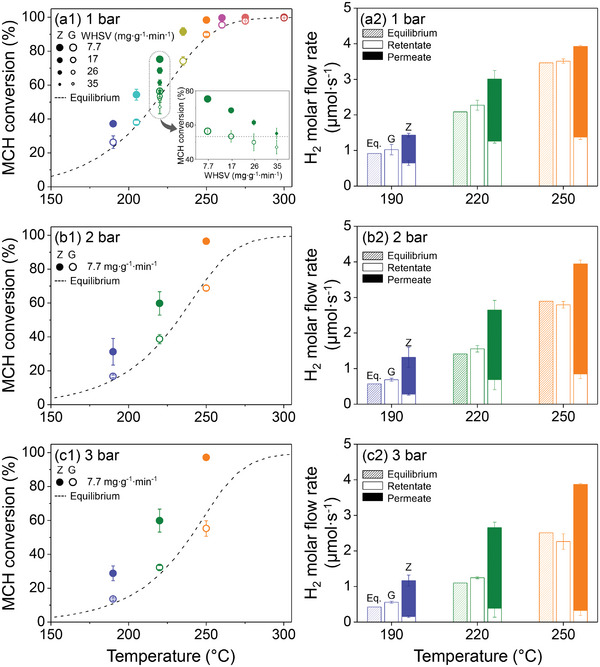
(a1)‐(c1) MCH conversions with a G‐based packed‐bed reactor (empty symbols) and Z‐based MR (filled symbols) and (a2)‐(c2) the corresponding H_2_ molar flow rates during MCH dehydrogenation reactions at (a1)‐(a2) 1, (b1)‐(b2) 2, and (c1)‐(c2) 3 bar at 190, 220, and 250 °C (WHSV of 7.7 mg g^−1^ min^−1^). In all graphs, the MCH conversions and corresponding H_2_ molar flow rates at equilibrium were calculated using Equation (S5)^[^ ^33^ ^]^ (Supporting Information). In (a1), MCH dehydrogenation reactions were conducted at WHSVs of 7.7, 17, 26, and 35 mg g^−1^ min^−1^ (220 °C; redrawn as inset) and temperatures of 190, 205, 220, 235, 250, 260, 275, and 300 °C. In (b1)‐(c1), the reaction was carried out at a WHSV of 7.7 mg g^−1^ min^−1^ at 190, 220, and 250 °C. Data shown here are averaged results of three independent samples and error bars (representing the corresponding standard deviations) are included for all data points. For clarity, the error bars that are embedded in the symbols (so not conspicuous) are owing to the low standard deviation values.

To elucidate the effect of the zeolite membrane on MR performance, MCH dehydrogenation reactions in a G‐based packed‐bed reactor (i.e., glazed impermeable wall in the MR configuration as a reference) and Z‐based MR were investigated at various WHSVs (7.7, 17, 26, and 35 mg g^−1^ min^−1^), reaction temperatures (190, 205, 220, 235, 250, 260, 275, and 300 °C), and total pressures on the feed side (1, 2, and 3 bar) (**Figure** **3**). For the G‐based packed‐bed reactor at 220 °C (Figure 3a1), the MCH conversion increased with the decrease in WHSV (green). Specifically, at a WHSV of 7.7 mg g^−1^ min^−1^, the MCH conversion only differed by ≈3% from the calculated equilibrium MCH conversion, indicating the achievement of the equilibrium state. In contrast, in the Z‐based MR configuration, a decrease in WHSV led to a significant increase in MCH conversion over the equilibrium curve because of the efficient removal of H_2_ from the product stream, thus shifting the equilibrium toward the product side. Furthermore, the MCH conversion at 1 bar increased with the increase in reaction temperature (from 190 to 250 °C) for both cases with Z and G, as expected for an endothermic reaction.

In particular, for the G‐based packed‐bed reactor, MCH conversion followed the equilibrium curve at each reaction temperature, indicating that the residence time was sufficient to achieve equilibrium. Notably, in the Z‐based MR configuration, the MCH conversion exceeded the thermodynamically limited equilibrium conversions at all temperatures, achieving high MCH conversions at moderate temperatures, with 98.3% ± 0.7% and 99.7% ± 0.1% at 250 and 260 °C, respectively, compared to the equilibrium conversions of 88.4% and 94.5% at the same respective temperatures and almost complete MCH conversions at higher temperatures (99.9% ± 0.01% and 100% ± 0.00% at 275 and 300 °C, respectively, vs 97.5% ± 0.3% and 99.7% ± 0.1% for the G‐based MR configuration at the same temperatures, respectively).

As shown in Figure 3b1‐c1, we performed additional MCH dehydrogenation reactions at higher total pressures on the feed side (2 and 3 bar). We considered three different reaction temperatures (190, 220, and 250 °C) as representative temperatures. In general, Le Chatelier's principle indicates that an increased total pressure in the MCH dehydrogenation reaction will favor a reverse reaction to the reactant side. Indeed, when the total pressure on the feed side increased from 1 to 3 bar at 250 °C, the MCH conversion with G decreased from 89.8% ± 1.0% to 55.2% ± 4.6% (Figure 3a1‐c1). However, in the Z‐based MR, an increase in the total pressure on the feed side increased the H_2_ molar flow rate to the permeate side proportionally (Figure 3a2‐c2) and, thus, shifted the equilibrium state to the product side, compensating for the preferred reverse reaction at high pressures (Figure 3a1‐c1). Consequently, the MCH conversion in the Z‐based MR did not change considerably (98.3% ± 0.7% at 1 bar vs 97.2% ± 1.7% at 3 bar at 250 °C). Furthermore, Figures S6–S8 (Supporting Information) show the molar flow rates of H_2_, Tol, and MCH in the product stream in the G‐ and Z‐based MRs at different total pressures (1, 2, and 3 bar) and reaction temperatures (190, 220, and 250 °C). The higher MCH conversion in the Z‐based MR suggests that higher molar flow rates of H_2_ and Tol and lower molar flow rates of MCH were obtained compared to those in the G‐based packed‐bed reactor.

### 2.4 Long‐Term Stability of the Z‐Based MR for MCH Dehydrogenation

**Figure 4:**
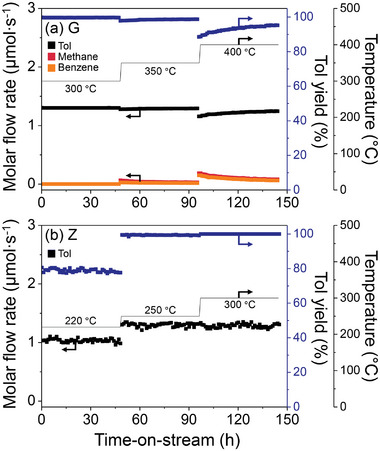
Molar flow rates of hydrocarbon products and Tol yields with the (a) G‐based packed‐bed reactor and (b) Z‐based MR as a function of time‐on‐stream during MCH dehydrogenation reactions. In (a), the reactions at each temperature (300, 350, or 400 °C) were conducted for 48 h, and different reaction temperatures (220, 250, and 300 °C) with the same time schedule were used in (b). In (b), only Tol was detected as a hydrocarbon product. For clarification, Tol, methane, and benzene are marked in black, red, and orange, respectively. Reaction condition: *P*_Total_ = 1 bar, WHSV = 7.7 mg g^−1^ min^−1^, flow rate of carrier gas (Ar) = 10 mL min^−1^, and flow rate of sweep gas (Ar) = 50 mL min^−1^.

Along with the initial MR performance, we tested the long‐term stability of the Z‐based MR at 220, 250, and 300 °C, as the robustness of MR is critical for practical uses. As a reference, we also performed MCH dehydrogenation reactions in the G‐based packed‐bed reactor at higher temperatures of 300, 350, and 400 °C (**Figure** **4**a). As expected from the equilibrium curve for an endothermic reaction (Figure 3a1), the Tol yields reached 99.7% ± 0.03% (close to 100%) at a relatively high temperature of 300 °C. However, the Tol yields decreased as the temperature increased above 350 °C because the unwanted demethylation of Tol occurred, yielding benzene and methane^[^ ^7^ ^]^: Additional information is given in Subsection S2.2 (Supporting Information).

A further increase in the reaction temperature (450 °C) resulted in activating the unwanted demethylation and producing the corresponding side products considerably (Figure S9, Supporting Information). Specifically, the Tol yield decreased from ≈99.4% ± 0.2% at 300 °C to 51.9% ± 3.1% at 450 °C. Therefore, a low working temperature in the MR, as shown in Figure 3 for the Z‐based MR configuration, is desirable for practical applications.

Generally, at low temperatures, catalytic MCH dehydrogenation activity decreased, and a relatively low Tol yield of ≈78.8% was obtained during long‐term stability tests at 220 °C (Figure 4b). However, at moderate temperatures (250–300 °C), the Tol yield reached ≈100%, which was maintained throughout the test without noticeable catalytic deactivation or side reactions. As expected from the robust zeolite membranes,^[^ ^34^ ^]^ the long‐term stabilities of the Z_out_‐based MR conducted at 220, 250, and 300 °C (Figures S10 and S11b, Supporting Information) were comparable to those of the Z‐based MR (Figure 4; Figure S11a, Supporting Information), indicating the effective use of zeolite membranes for the MR configuration. Unless all identical parameters are considered, a direct comparison of performance is not feasible. Nevertheless, prolonged exposure beyond the duration time adopted in this study (i.e., 48 h) may potentially lead to catalyst deactivation at a high temperature of over 300 °C.^[^ ^10^, ^35^ ^]^ Instead, a Tol yield of ≈99% at a lower temperature (e.g., 250 °C as seen in Figure 4b) was highly optimal to meet both important aspects of high MCH conversion (i.e., Tol yield) and catalyst stability, making the Z‐based MR industrially available.^[^ ^36^ ^]^ Indeed, simulation works indicated that for MCH‐based H_2_ production scale in the range of 30–700 m^3^ h^−1^, the MR configuration achieved cost reduction as high as 20.3−22.9%, compared to the conventional packed‐bed reactor configuration, and was economically feasible for real uses.^[^ ^15^ ^]^ Such benefits were consistently reported with MRs for other types of reactions.^[^ ^37^ ^]^ Furthermore, as shown in Figure S11 (Supporting Information), the molar flow rates of H_2_ on the permeate and retentate sides were almost constant, indicating the stable separation ability of both Z and Z_out_ for up to 7 d. In summary, at a moderate temperature (250 °C), the Z‐based MR avoided side reactions during MCH dehydrogenation and showed good reaction performance and durability. Notably, the current study will serve as a cornerstone to provide the technical data that can be used to estimate and determine the economic feasibility of the MR configuration in real applications,^[^ ^15^, ^37^ ^]^ which is, in turn, highly desirable for providing valuable insights into the MR‐based advancement and realization of the hydrogen economy.

### 2.5 Effects of the Total Pressure of the Permeate Side on MR Performance

**Figure 5:**
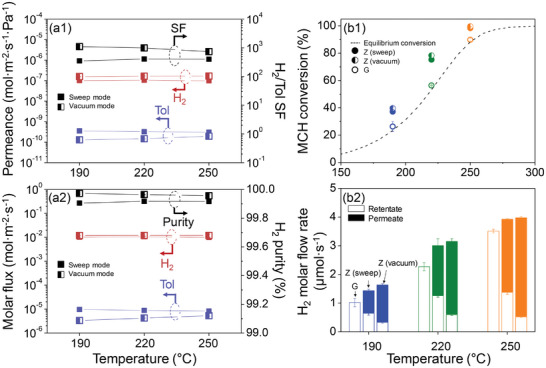
(a1) H_2_ and Tol permeances and H_2_/Tol SFs through Z and (a2) the corresponding H_2_ and Tol molar fluxes and H_2_ purities measured at a H_2_/Tol molar ratio of 3:1 in the mixed feed in vacuum mode (half‐filled symbols) and sweep mode (filled symbols). The results obtained in sweep mode are identical to those shown in Figure 2a1‐a2. (b1) MCH conversions and (b2) the corresponding H_2_ molar flow rates during MCH dehydrogenation with G‐based packed‐bed reactor and Z‐based MR in sweep and vacuum modes at different reaction temperatures (i.e., 190, 220, and 250 °C) while keeping the pressure of the feed side at 1 bar. In (b1), the MCH conversion curve at equilibrium was calculated using Equation (S5) (Supporting Information) and is marked by a gray dashed curve. Reaction condition: *P*_Total_ = 1 bar, WHSV = 7.7 mg g^−1^ min^−1^,and flow rate of carrier gas (Ar) = 10 mL min^−1^. Data shown here are averaged results of three independent samples and error bars (representing the corresponding standard deviations) are included for all data points. For clarity, the error bars that are embedded in the symbols (so not conspicuous) are owing to the low standard deviation values.

We further investigated the separation performance of Z in vacuum mode, considering any changes in the permeance, molar flux, H_2_/Tol SF, and H_2_ purity, as well as MCH dehydrogenation reaction performance. The enhanced permeation of H_2_ through the membrane in vacuum mode led to a significant improvement in all membrane properties (i.e., H_2_/Tol SFs of Z and, accordingly, H_2_ purity on the permeate side in **Figure** **5**a1‐a2) compared to those in sweep mode (Figure 2a1‐a2).

For comparison, the results in Figure 2a1‐a2 are included in Figure 5a1‐a2 (for clarity, the real values of permeances of H_2_/Tol through Z and their separation factors in vacuum mode are tabulated in Table S5 (Supporting Information), where the information of the corresponding H_2_ molar fluxes and purities is given as well). Notably, the maximum H_2_/Tol SF in vacuum mode at 190 °C was as high as ≈1105 ± 124 with a corresponding H_2_ purity of ≈99.97% ± 0.01%.

Moreover, the MCH dehydrogenation reaction performance in the Z‐based MR in vacuum mode led to a more effective shift in equilibrium to the product side than that in sweep mode (Figure 5b1‐b2), because of the enhanced permeation rate of H_2_ to the permeate side (Figure 5a1‐a2). In addition, the molar flow rates of H_2_, Tol, and MCH in the product stream at 190, 220, and 250 °C are shown in Figure S12 (Supporting Information). It was noted that stable molar flow rates of all the reactants (MCH) and products (H_2_ and Tol) were observed throughout the reaction up to 190 min, indicating the robustness of the MR configuration.

### 2.6 Evaluation of Z‐Based MR Performance for MCH Dehydrogenation

**Figure 6:**
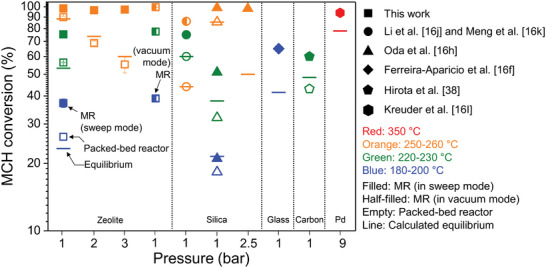
MCH conversion of the zeolite MR in the current study and those of other MRs reported in the literature.^[^ ^16^, ^38^ ^]^ For clarification, the membrane separation modes used in the MR are indicated by filled (sweep mode) and half‐filled (vacuum mode) symbols. For comparison, the MCH conversion in the conventional packed‐bed reactor (empty symbols) and in the equilibrium state (marked by lines) are shown. The types of membranes used for the MR configuration are indicated at the bottom. The colors of the symbols represent the reaction temperatures (blue: 180–200 °C, green: 220–230 °C, orange: 250–260 °C, and red: 350 °C). Data (square) obtained in this work are averaged results of three independent samples and error bars (representing the corresponding standard deviations) are included for all data points. For clarity, the error bars that are embedded in the symbols (so not conspicuous) are owing to the low standard deviation values.

A literature survey revealed that silica,^[^ 16h,^j,k^ ^]^ glass,^[^ ^16f^ ^]^ carbon,^[^ ^38^ ^]^ and Pd^[^ ^16l^ ^]^ membranes have been used in the MR configuration for MCH dehydrogenation because of their high H_2_ separation capabilities. Although we would like to compare the enhanced MCH conversion in the Z‐based MR (e.g., Figure 3 in this study) with others in the literature, to the best of our knowledge, an evaluation protocol for MCH dehydrogenation reaction performance in the MR configuration has not been reported yet. Therefore, we carried out an evaluation and comparison of results obtained using different MRs reported in the literature with those of our MR, as shown schematically in **Figure** **6**. As shown, the Z‐based MR provided remarkable performance at temperatures of 190–250 °C and pressures of 1–3 bar.

Specifically, the MR with both the zeolite (i.e., Z in this work) and silica membranes showed significant improvements in MCH conversion, exceeding equilibrium conversion at moderate temperatures (≈220–230 and 250–260 °C), above which the endothermic MCH dehydrogenation reaction will be thermally activated (e.g., ≥300 °C^[^ ^10b^ ^]^). Although MRs based on glass, carbon, and Pd membranes also shift equilibrium to the product side, the differences between the MCH conversion values obtained in the MR configuration and the corresponding equilibrium conversion values were less notable than those of the MRs with Z and silica membranes. This suggests that the MRs with Z and silica membranes outperformed those with glass, carbon, and Pd membranes in terms of enhancing the MCH conversion. Nevertheless, it was noted that the Z‐based MR yielded higher MCH dehydrogenation performance at low temperatures (180–200 °C) than silica‐membrane‐based MR, seemingly because of the high rate of H_2_ permeation through Z in this study. Although the single‐component H_2_ permeance through the silica membranes was almost constant from 100 to 300 °C,^[^ ^16h^ ^]^ the H_2_ permeance in the presence of Tol in the feed was considerably reduced at low temperatures because of the physical adsorption of toluene molecules on the membrane surface or inside the pores, as reported in the literature.^[^ ^39^ ^]^ Consequently, the reaction did not shift substantially toward the forward reaction. In contrast, Z showed almost temperature‐independent separation performance for H_2_/Tol mixtures (Figure 2a). Therefore, even at low reaction temperatures, the H_2_ permeance through Z in the MR configuration was not hindered by Tol adsorption onto the membrane, and its high permeance ability was maintained, suggesting a positive effect of high‐performance membrane separation ability on the degree of equilibrium shift in the MR configuration for MCH dehydrogenation.

In particular, the robustness of zeolites makes Z suitable for use for MCH dehydrogenation, as compared to the conventional H_2_‐permselective silica membranes. For example, the pores of pure silica membranes shrink at high temperatures as a result of the thermal condensation of the Si–OH groups.^[^ ^40^ ^]^ Further, some silica membranes having organic moieties are not stable above 400 °C because of the degradation or combustion of the organics.^[^ ^16d^ ^]^ Although high‐temperature stability tests are needed for fair evaluation and comparison, these phenomena would make silica membranes less competitive for long‐term use. In contrast, zeolite membranes have a well‐defined crystalline structure and are purely inorganic, resulting in thermal stability up to 500 °C.^[^ ^41^ ^]^ Therefore, thermally stable zeolite membranes are a good option for high‐temperature processes such as MCH dehydrogenation.

## 3 Conclusion

In this study, we synthesized a DDR@CHA hybrid zeolite membrane using a heteroepitaxial growth method and, further, used it in MR configuration for MCH dehydrogenation. We also investigated the effect of mass transfer between the packed catalyst and the zeolite membrane in MR configuration on MCH reaction activity. The Z membrane (i.e., a DDR zeolite membrane grown from a CHA seed layer on the inner surface of the tubular support) was similar to Z_out_ (prepared on the outer surface) in terms of membrane morphology and crystallinity. Z in MR configuration resulted in more improvement of MCH conversion than Z_out_, indicating the effective H_2_ removal during MCH dehydrogenation. In particular, the separation performance of Z for H_2_/Tol and H_2_/MCH mixtures was remarkable, yielding the corresponding SFs as high as ≈380 and 460, respectively, at 190–300 °C. The use of Z as walls in MR configuration for MCH dehydrogenation shifted equilibrium to the product side via the selective removal of H_2_ through the membranes in sweep mode, thus overcoming the thermodynamic limit. Accordingly, the MR configuration with Z (can achieve a high H_2_ purity of ≈99% or higher) exceeded the MCH equilibrium conversion and purified H_2_ on the permeate side. Notably, the use of Z in MR configuration resulted in considerably improved MCH conversion under all studied conditions, which is *T* = 190–300 °C, *P* = 1–3 bar, and WHSV = 7.7–35 mg g^−1^ min^−1^. In one case, the MCH conversion in MR configuration was 75.3% ± 0.8% at 220 °C, significantly higher than that (56.4% ± 0.3%) of a conventional packed‐bed reactor, with the H_2_ purity of 99.7% ± 0.2% on the permeate side.

Crucially, the use of a working temperature below 300 °C avoided side product formation (here, demethylation of Tol) and, thus, increased the H_2_ purity while producing only Tol as a H_2_‐lean LOHC. Further, the Z‐based MR configuration exhibited robust performance, suggesting its practical potential. In addition, an enhanced equilibrium shift to the product side was achieved, apparently owing to facilitated H_2_ permeation through the membrane in vacuum mode. Notably, we confirmed that the Z‐based MR configuration enhanced MCH dehydrogenation performance at low reaction temperatures, and this performance was comparable to those achieved using MRs that exploited other membrane materials (especially silica). Thus, the H_2_‐permselective zeolite membranes are a promising option for enhancing the low‐temperature catalytic activity of MCH dehydrogenation through selective H_2_ separation from the product stream. As a follow‐up task, we would like to use a membrane module containing multiple zeolite membranes to increase the H_2_ production rate and enhance the scope for practical applications.

## Conflict of Interest

The authors declare no conflict of interest.

## Supporting information

Supporting Information

## Data Availability

The data that support the findings of this study are available from the corresponding author upon reasonable request.

## References

[1] a) H. Nishiyama , T. Yamada , M. Nakabayashi , Y. Maehara , M. Yamaguchi , Y. Kuromiya , Y. Nagatsuma , H. Tokudome , S. Akiyama , T. Watanabe , R. Narushima , S. Okunaka , N. Shibata , T. Takata , T. Hisatomi , K. Domen , Nature 2021, 598, 304; 34433207 10.1038/s41586-021-03907-3

[2] a) X. F. Lu , S. L. Zhang , W. L. Sim , S. Y. Gao , X. W. Lou , Angew. Chem., Int. Ed. 2021, 60, 22885; 10.1002/anie.202108563 34351663

[3] a) S. E. Hosseini , M. A. Wahid , Int. J. Energy Res. 2020, 44, 4110;

[4] a) K. Grubel , H. Jeong , C. W. Yoon , T. Autrey , J. Energy Chem. 2020, 41, 216;

[5] a) D. Teichmann , W. Arlt , P. Wasserscheid , R. Freymann , Energy Environ. Sci. 2011, 4, 2767;

[6] L. Han , L. J. Zhang , H. Wu , H. L. Zu , P. X. Cui , J. S. Guo , R. H. Guo , J. Ye , J. F. Zhu , X. S. Zheng , L. Q. Yang , Y. C. Zhong , S. Q. Liang , L. B. Wang , Adv. Sci. 2019, 6, 1900006. 10.1002/advs.201900006 PMC6662073 31380161

[7] F. Alhumaidan , D. Tsakiris , D. Cresswell , A. Garforth , Int. J. Hydrogen Energy 2013, 38, 14010.

[8] a) N. Kariya , A. Fukuoka , T. Utagawa , M. Sakuramoto , Y. Goto , M. Ichikawa , Appl. Catal. A 2003, 247, 247;

[9] a) X. T. Zhang , N. He , L. Lin , Q. R. Zhu , G. Wang , H. C. Guo , Catal. Sci. Technol. 2020, 10, 1171;

[10] a) F. Alhumaidan , D. Cresswell , A. Garforth , Ind. Eng. Chem. Res. 2010, 49, 9764;

[11] Y. Yuan , Z. Q. Zhao , R. F. Lobo , B. J. Xu , Adv. Sci. 2023, 10, 2207756. 10.1002/advs.202207756 PMC10161086 36897033

[12] a) A. H. Al‐ShaikhAli , A. Jedid , D. H. Anjum , L. Cavallo , K. Takanabe , ACS Catal. 2017, 7, 1592;

[13] a) H. Z. Li , C. L. Qiu , S. J. Ren , Q. B. Dong , S. X. Zhang , F. L. Zhou , X. H. Liang , J. G. Wang , S. G. Li , M. Yu , Science 2020, 367, 667; 32029624 10.1126/science.aaz6053

[14] a) S. H. Morejudo , R. Zanon , S. Escolastico , I. Yuste‐Tirados , H. Malerod‐Fjeld , P. K. Vestre , W. G. Coors , A. Martinez , T. Norby , J. M. Serra , C. Kjolseth , Science 2016, 353, 563; 27493179 10.1126/science.aag0274

[15] M. Byun , H. Kim , C. Choe , H. Lim , Energy Convers. Manag. 2021, 227, 113576.

[16] a) J. K. Ali , E. J. Newson , D. W. T. Rippin , Chem. Eng. Sci. 1994, 49, 2129;

[17] J. Melendez , N. de Nooijer , K. Coenen , E. Fernandez , J. L. Viviente , M. V. Annaland , P. L. Arias , D. A. P. Tanaka , F. Gallucci , J. Membr. Sci. 2017, 542, 329.

[18] a) H. L. Song , Y. Peng , C. L. Wang , L. Shu , C. Y. Zhu , Y. L. Wang , H. Y. He , W. S. Yang , Angew. Chem., Int. Ed. 2023, 62, e202218472; 10.1002/anie.202218472 36854948

[19] a) P. Du , J. Y. Song , X. R. Wang , Y. T. Zhang , J. X. Xie , G. Liu , Y. L. Liu , Z. W. Wang , Z. Hong , X. H. Gu , J. Membr. Sci. 2021, 636, 119546;

[20] R. S. Xu , L. He , L. Li , M. J. Hou , Y. Z. Wang , B. S. Zhang , C. H. Liang , T. H. Wang , J. Energy Chem. 2020, 50, 16.

[21] M. Kanezashi , K. Yada , T. Yoshioka , T. Tsuru , J. Am. Chem. Soc. 2009, 131, 414. 19113940 10.1021/ja806762q

[22] a) Z. P. Lai , G. Bonilla , I. Diaz , J. G. Nery , K. Sujaoti , M. A. Amat , E. Kokkoli , O. Terasaki , R. W. Thompson , M. Tsapatsis , D. G. Vlachos , Science 2003, 300, 456; 12624179 10.1126/science.1082169

[23] M. J. den Exter , J. C. Jansen , H. van Bekkum , Stud. Surf. Sci. Catal. 1994, 84, 1159.

[24] Y. Jin , Y. Y. Fan , X. X. Meng , W. M. Zhang , B. Meng , N. T. Yang , S. M. Liu , Processes 2019, 7, 751.

[25] H. H. Funke , A. M. Argo , J. L. Falconer , R. D. Noble , Ind. Eng. Chem. Res. 1997, 36, 137.

[26] a) Y. Jeong , S. Hong , E. Jang , E. Kim , H. Baik , N. Choi , A. C. K. Yip , J. Choi , Angew. Chem., Int. Ed. 2019, 58, 18654; 10.1002/anie.201911164 31591796

[27] a) R. Raso , M. Tovar , J. Lasobras , J. Herguido , I. Kumakiri , S. Araki , M. Menendez , Catal. Today 2021, 364, 270;

[28] E. Kim , W. X. Cai , H. Baik , J. Nam , J. Choi , Chem. Commun. 2013, 49, 7418. 10.1039/c3cc42779j 23851641

[29] a) S. W. Yang , B. Min , Q. Fu , C. W. Jones , S. Nair , Angew. Chem., Int. Ed. 2022, 61, e202204265; 10.1002/anie.202204265 35536251

[30] a) K. Kida , Y. Maeta , T. Kuno , K. Yogo , Chem. Lett. 2017, 46, 1724;

[31] a) F. Kapteijn , X. R. Wang , Chem. Ing. Tech. 2022, 94, 23;

[32] a) M. Y. Cao , L. H. Zhao , D. W. Xu , R. Ciora , P. K. T. Liu , V. I. Manousiouthakis , T. T. Tsotsis , J. Membr. Sci. 2020, 605, 118028;

[33] T. Schildhauer , E. Newson , S. Muller , J. Catal. 2001, 198, 355.

[34] a) N. Goswami , A. Bose , N. Das , S. N. Achary , A. K. Sahu , V. Karki , R. C. Bindal , S. Kar , Int. J. Hydrogen Energy 2017, 42, 10867;

[35] a) P. Ferreira‐Aparicio , I. Rodríguez‐Ramos , A. Guerrero‐Ruiz , Chem. Commun. 2002, 2082; 10.1039/b205300d 12357789

[36] a) D. D. Papadias , J. K. Peng , R. K. Ahluwalia , Int. J. Hydrogen Energy 2021, 46, 24169;

[37] a) S. Kim , S. W. Yun , B. Lee , J. Heo , K. Kim , Y. T. Kim , H. Lim , Int. J. Hydrog. Energy 2019, 44, 2330;

[38] Y. Hirota , A. Ishikado , Y. Uchida , Y. Egashira , N. Nishiyama , J. Membr. Sci. 2013, 440, 134.

[39] M. Seshimo , K. Akamatsu , S. Furuta , S. Nakao , Sep. Purif. Technol. 2015, 140, 1.

[40] a) S. O. Lawal , H. Nagasawa , T. Tsuru , M. Kanezashi , J. Membr. Sci. 2022, 642, 119948; 10.3390/membranes13010030 PMC9860565 36676837

[41] H. B. Wang , X. L. Dong , Y. S. Lin , J. Membr. Sci. 2014, 450, 425.

