# Supplementary material for: Zeolite Membrane‐Based Low‐Temperature Dehydrogenation of a Liquid Organic Hydrogen Carrier: A Key Step in the Development of a Hydrogen Economy
Source: Adv Sci (Weinh). 2024 Jun 13;11(30):2403128. doi: 10.1002/advs.202403128 (PMC11321665; doi:10.1002/advs.202403128)
Supplement: Supplementary file 1 — Supporting Information [file ADVS-11-2403128-s001.pdf]

ADVANCED SCIENCE
Open Access

Supporting Information

for *Adv. Sci.*, DOI 10.1002/advs.202403128

Zeolite Membrane-Based Low-Temperature Dehydrogenation of a Liquid Organic Hydrogen Carrier: A Key Step in the Development of a Hydrogen Economy

Sejin Kim, Seungmi Lee, Suhyeon Sung, Sangseo Gu, Jinseong Kim, Gihoon Lee, Jaesung Park, Alex C. K. Yip and Jungkyu Choi**

Supporting Information

Zeolite Membrane-Based Low-Temperature Dehydrogenation of a Liquid Organic Hydrogen Carrier: A Key Step in the Development of a Hydrogen Economy

Sejin Kim,[1,†] Seungmi Lee,[1,†] Suhyeon Sung,[1,†] Sangseo Gu,[1] Jinseong Kim,[1]

Gihoon Lee,[1] Jaesung Park,[2] Alex C. K. Yip,[3,*] and Jungkyu Choi[1,*]

[1] Department of Chemical and Biological Engineering, Korea University, 145 Anam-ro, Seongbuk-gu, Seoul 02841, Republic of Korea

[2] Green Carbon Research Center, Korea Research Institute of Chemical Technology (KRICT), Daejeon 34114, Republic of Korea

[3] Chemical and Process Engineering, University of Canterbury, Christchurch 8140, New Zealand

[†] These equally contributed to this work.

* Corresponding authors

(ACKY) E-mail address: alex.yip@canterbury.ac.nz and Phone: +64-3-369-4086

(JC) E-mail address: jungkyu_choi@korea.ac.kr, Tel: +82-2-3290-4854, and Fax: +82-2-926-6102

S1. Experimental

S1.1. Synthesis of CHA particles and formation of CHA seed layers on the inner surface of tubular supports

Asymmetric α-alumina tubular supports (OD: 12 mm, ID: 8 mm, length: 90 mm, porosity: 39%, and mean pore diameter: 0.86 μm; FINETECH Co., Ltd., Republic of Korea) were used to fabricate DDR@CHA hybrid zeolite membranes (for convenience, CHA and DDR type zeolites are denoted CHA and DDR, respectively). Both ends of the tubular support (approximately 2 cm), which were used for sealing the cell, were glazed with impermeable materials (IN1001 Envision Glazes, Duncan Ceramics, USA) before the synthesis of the zeolite membrane. After glazing, CHA seed particles (Si/Al ratio of approximately 20 ± 2) were deposited on the inner surface. The synthesis of CHA seed particles has been described previously.[1] Briefly, *N*,*N*,*N*-trimethyl-1-adamantylammonium hydroxide (TMAdaOH, 25 wt%, and product number: T3821, SACHEM, Inc.) and sodium hydroxide (NaOH, ≥ 98%, and product number: S5881, Sigma-Aldrich) were added to deionized (DI) water in a 250-mL polypropylene (PP) bottle. The mixture was homogenized on a shaker for approximately 20 min. Then, a silica source (LUDOX® HS-40 colloidal silica, 40 wt% suspension in H_2O, and product number: 420816, Sigma-Aldrich) was added dropwise to the solution while stirring. The resulting mixture was stirred at room temperature for 48 h. The final molar composition was 100 SiO_2: 20 NaOH: 5 $Al(OH)_3$: 20 TMAdaOH: 1600 H_2O. After stirring, the mixture was transferred to a Teflon liner (ca. 45 mL) in a stainless-steel autoclave. The autoclaves were moved to an oven (PL-HC_250, Pluskolab, Republic of Korea) preheated to 160 °C and rotated at 45 rpm during hydrothermal reaction. After 7 d of hydrothermal reaction, the autoclaves were quenched with tap water to stop the reaction, and the synthesized particles were recovered by repeating a series of centrifugation, decanting, and redispersion in DI water five times. The recovered particles were dried in an oven at 70 °C overnight and then calcined at 550 °C for 12 h at a ramp rate of 1 $°C \cdot min^{-1}$ in air (flow rate: 200 $mL \cdot min^{-1}$) in a box furnace (CRF-M20-UP, Pluskolab, Republic of Korea). Next, the CHA particles were deposited by dip-coating the inner surface of a tubular support (dip coater: ZID-6A,

Jaesung Engineering Co., Republic of Korea). For this, the CHA seed particles were added to ethanol in the PP bottle and, then, the mixture was sonicated (UC-10, JeioTech Co. Ltd., Republic of Korea) for 20 min to achieve a good dispersion. The concentration of the CHA suspension was ca. 0.75 $g_{seed\ particles} \cdot L_{ethanol}^{-1}$. For dip-coating, the seed suspension (ca. 50 mL) was transferred to a 50-mL graduated cylinder. The tubular support was moved vertically downward to immerse it completely in the seed suspension. After immersion for ca. 30 s, the tubular support was lifted to its initial position and dried for ca. 30 s under ambient conditions. This dip-coating process was repeated fourteen times to obtain a uniform and dense seed layer. After dip-coating seven times in one direction, the tubular support was turned upside down and dip-coated seven more times. After dip-coating was complete, the seed particle-deposited tubular support was removed from the dip coater and dried at room temperature for ca. 30 min. Finally, the seeded tubular support was calcined at 450 °C for 4 h at a ramp rate of 1 $°C \cdot min^{-1}$ in a box furnace.

S1.2. Synthesis of a DDR membrane from the CHA seed layer

Conventional DDR@CHA hybrid membranes (denoted Z_{out}) were synthesized using our previous method.[2] In particular, the Z_{out} membrane was used to compare the properties and performance of the membranes (denoted Z) grown on the inner surface of the tubular support in this study. The DDR synthetic precursor was also prepared using a reported method.[3] First, ethylenediamine (EDA, ≥ 99%, and product number: E26266, Sigma-Aldrich) and 1-adamantlyamine (ADA, 98%, and product number: H30076, Alfa Aesar), an organic structure-directing agent (OSDA) for DDR synthesis, were added to a PP bottle sequentially and homogenized by 20-min sonication. Subsequently, DI water was quickly added to the mixture, resulting in an opaque mixture, which was shaken for 1 h and, then, placed in a preheated oil bath at 95 °C. The mixture was magnetically stirred for more than 3 h until clear. Then, the clear solution was moved to an ice water bath and magnetically stirred for 20 min. After cooling, fumed silica (CAB-O-SIL M5, Cabot Corp., USA) was added to the mixture and shaken under ambient conditions for 12 h. The

final molar composition of the all-silica DDR synthetic precursor was 100 SiO_2: 47 ADA: 404 EDA: 11240 H_2O.

The DDR membrane was synthesized from the CHA seed layer as follows. The DDR synthetic precursor (90 mL) was poured into a Teflon liner (total volume: ca. 120 mL) containing the seeded tubular support. The Teflon liner was sealed in a stainless-steel autoclave and moved to a convection oven (PL_HV_250, Pluskolab, Republic of Korea), preheated to 160 °C. The hydrothermal reactions were conducted under static conditions for 1 d. The autoclave was quenched with tap water to stop the hydrothermal reaction. After cooling, the as-synthesized membrane on the tubular support was taken out and the outer surface of the tubular support was ground with sandpaper to remove any grown zeolite. The membrane was then washed in a 500-mL beaker filled with DI water for 12 h. The sample was dried in a drying oven (HB-502M, Pluskolab, Republic of Korea) at 70 °C. Finally, the dried tubular zeolite membrane was calcined in a quartz tube (OD: 50 mm and ID: 46 mm) in flowing ozone at 250 °C for 40 h at a ramp rate of 0.2 $°C \cdot min^{-1}$ in a tubular furnace (Scientech, Republic of Korea). The ozone stream comprised 5 vol% ozone balanced with pure oxygen. The ozone stream was generated by flowing a pure oxygen gas flow (99.9% purity) at 1000 $mL \cdot min^{-1}$ through an ozone generator (OZE-020, Ozone Engineering Co., Ltd., Republic of Korea). Finally, to compare the effects of H_2 separation on the MCH dehydrogenation reaction, an impermeable tubular support was fabricated by glazing the entire outer surface of the tubular support with the aforementioned glazing material. For convenience, the impermeable membrane prepared on the tubular support is denoted G.

S1.3. Characterization

Field-emission scanning electron microscopy (FE-SEM; S-4800, Hitachi Ltd., Japan) images of the membrane samples were acquired by sputter-coating (E-1045, Hitachi Ltd., Japan) the samples with Pt at 30 mA for 30 s. X-ray diffractometry (XRD; D/Max-2500V/PC X-ray diffractometer, Rigaku Co., Japan) measurements were carried out with Cu K_α radiation (λ = 0.154 nm). For comparison, simulated

XRD patterns of the all-silica CHA and DDR zeolites were generated using Mercury (available from the website of Cambridge Crystallographic Data Centre; https://www.ccdc.cam.ac.uk). The corresponding crystallographic information files were downloaded from the International Zeolite Association (IZA) website (http://www.iza-online.org).

S1.4. Separation performance of DDR@CHA hybrid membranes

Considering that the ratio of stoichiometric coefficients of H_2 and Tol in the MCH dehydrogenation reaction is 3:1, the H_2/Tol separation abilities of the prepared hybrid zeolite membranes (Z) were evaluated using a feed mixture with a molar ratio of 3:1. Similarly, in the H_2/MCH separation tests, the feed mixture had a molar ratio of 3:1. The performance of Z was assessed in terms of permeance, separation factor, and molar flux at temperatures from 190 to 300 °C. Argon (Ar) gas was used as a sweep gas at a flow rate of 50 $mL{\cdot}min^{-1}$ to ensure proper separation performance measurements and the total pressure of the permeate side was maintained at 1 bar. In addition, the H_2/Tol separation performance tests were conducted at different total feed pressures (1-3 bar) at a constant temperature (220 °C). During the separation tests, the H_2 gas molar flow rates on the retentate and permeate sides were measured using an online gas chromatograph (GC, YL6500, YL Instrument Co., Ltd.) equipped with a thermal conductivity detector (TCD) and the Tol and MCH flow rates on both sides were measured using the online GC equipped with a flame ionization detector (FID). In this study, methane (10 $mL{\cdot}min^{-1}$) was used as an internal standard for the reliable GC analyses of the molar compositions on the retentate and permeate sides. To account for the concentration gradient along the axial direction of the tubular membrane, the log-mean average pressure drop was used to calculate the permeance accurately.

S1.5. Membrane reactor for MCH dehydrogenation

The setups of the MR and packed-bed reactor configurations are shown in Scheme S1. Zeolite (Z and Z_{out}) and impermeable (G) membranes were used for the MR and packed-bed reactor, respectively. The MCH dehydrogenation reaction was performed using pelletized catalysts (5 wt% Pt on activated

carbon (Pt/C) in the range of 150-250 µm and product number: 19523, Alfa Aesar) with quartz sand (in the range of 200-300 µm and product number: 274739, Sigma-Aldrich). To fill the inner space of a tubular membrane sample (i.e., Z, Z_{out}, and G) with catalyst, quartz wool was placed at the bottom and a mixture of Pt/C and quartz sand was added. Quartz wool was also placed at the top to fix the mixed particles. For comparison, the MCH dehydrogenation reaction was also conducted in a quartz reactor (denoted Q; OD: 9.6 mm and ID: 6.9 mm). Here, pelletized Pt/C particles were loaded on the quartz wool in the middle of the quartz tube reactor. For the MRs, the MCH feed (0.01 $mL \cdot min^{-1}$ as a liquid phase) carried by Ar was continuously fed into the reactor. For reliability, the MCH liquid was injected by a high-pressure pump (SP930D, YL Instrument Co., Ltd., Republic of Korea) and evaporated by heating the tube lines at 200 °C using a temperature controller (UP35A, Yokogawa, Japan). In sweep mode, Ar was used as both carrier (10 $mL \cdot min^{-1}$) and sweep gas (50 $mL \cdot min^{-1}$) using a mass flow controller (MFC, High Tech, Bronkhorst). The reaction temperature and pressure of the feed side were controlled using a temperature controller with a thermocouple inside the furnace and a back pressure regulator, respectively. A weight hourly space velocity (WHSV) was obtained by dividing the mass flow rate of MCH (7.7 $mg \cdot min^{-1}$) by the mass of Pt/C and tuned by changing the amount of Pt/C (in this study, catalyst weights of 0.222, 0.3, 0.461, and 1 g correspond to WHSVs of 35, 26, 17, and 7.7 $mg \cdot g^{-1} \cdot min^{-1}$, respectively). During MCH dehydrogenation, the molar flow rates of MCH and products (H_2 and Tol) on the retentate and permeate sides were measured as described in Subsection S1.4. For high-temperature experiments (i.e., $\geq$ 350 °C), propane (10 $mL \cdot min^{-1}$) was used as the internal gas instead of methane, which was produced as a by-product under these conditions. MCH dehydrogenation reactions were conducted three times to ensure reliability.

For performance assessment, MCH conversion and Tol yield were calculated using Equations (S1) and (S2), respectively:

$$\mathrm{MCH\ conversion}(\%) = \frac{F_{MCH,in} - F_{MCH,out}}{F_{MCH,in}} \times 100 \quad \text{and} \tag{S1}$$

$$\mathrm{Tol\ yield}(\%) = \frac{F_{Tol,out}}{F_{MCH,in}} \times 100 \tag{S2}$$

where $F_{MCH,\mathrm{in}}$ is the molar flow rate of MCH in the inlet stream fed to the reactor and $\mathrm{F}_{MCH,\ \mathrm{out}}$ and $F_{Tol,\mathrm{out}}$ are the molar flow rates of MCH and Tol in the outlet stream exiting the reactor, respectively. For reference, the equilibrium MCH conversions were calculated as follows. First, the reaction rate is given by Equation (S3):

$$r = k \cdot p_{MCH} \cdot \left\{ 1 - \left(\frac{p_{Tol} \cdot p_{H_2}^3}{K_{eq,T} \cdot p_{MCH}} \right) \right\} \tag{S3}$$

where r, k, p_i, and $K_{eq,T}$ are the reaction rate, reaction rate constant, partial pressures of the components (i = MCH, Tol, or H_2), and equilibrium constant, respectively. In particular, the equilibrium constants ($K_{eq,\ T}$) at a given temperature (T) can be calculated based on the Van't Hoff equation (Equation (S4)),

$$K_{eq,T} = K_{eq,\ 650[\mathrm{K}]} \cdot \exp\left\{ \frac{217\,650\,[\mathrm{J \cdot mol^{-1}}]}{R} \left(\frac{1}{T} - \frac{1}{650[K]} \right) \right\} \tag{S4}$$

where the equilibrium constant at 650 K ($K_{eq,\ 650\ [\mathrm{K}]}$: 3600 bar^3), which is a pre-exponential factor, was adopted from the literature.[4] The reaction rate is zero at equilibrium; thus, Equation (3) was used to obtain the equilibrium constant (Equation (S5)),

$$K_{eq,T} = \frac{27 p_{MCH,in}^3 \cdot X_{MCH}^4}{(1 - X_{MCH})(1 + 3 p_{MCH,in} \cdot X_{MCH})^3}. \tag{S5}$$

Consequently, the equilibrium MCH conversion (X_{MCH}) was calculated as a function of temperature and used as a reference.

S1.6. H_2/Tol separation performance of DDR@CHA hybrid membranes in vacuum mode and MR configuration for MCH dehydrogenation

In addition to sweep mode, vacuum mode was used to measure the H_2/Tol separation performance of Z and carry out MCH dehydrogenation reactions in the MR. For this, the total pressure on the permeate side was approximately 0.03 bar, achieved using a vacuum pump (N840G, KNF, Germany) and the total feed pressure was maintained at 1 bar. Other operating parameters for H_2/Tol separation and MCH dehydrogenation were the same as those used in sweep mode.

S2. Supplemental results and discussion

S2.1. Separation performance of Z and Z_{out} for CO_2/CH_4 mixtures

The CO_2/CH_4 separation performance of Z and Z_{out} was investigated according to the separation measurement protocol described in our previous study.[2] Specifically, the partial pressures of the CO_2/CH_4 binary mixture were approximately 50 kPa/50 kPa at a total feed rate of 1000 $mL \cdot min^{-1}$ in vacuum mode. Approximately, the total feed and permeate pressures were maintained at ca. 1 and 0.03 bar, respectively. A vacuum pump (DTC-22B, Ulvac Technologies Inc., Japan) was used to maintain low pressure on the permeate side. The log-mean average pressure drop was applied to calculate the permeance accurately, accounting for the concentration gradient along the axial direction of the tubular membrane. A permeation cell in which Z or Z_{out} was mounted in an oven (DX330, Yamato Scientific Co., Ltd., Japan) was to measure the CO_2/CH_4 separation performance at different temperatures. An online gas chromatograph (GC, YL 6500 GC, YL Instrument Co., Ltd.) equipped with a thermal conductivity detector (TCD) was used to analyze the molar composition of the molecules on the permeate side. Here, the permeating molecules were continuously carried to the GC via the vacuum pump. N_2 (ca. 10 $mL \cdot min^{-1}$) was used as an internal standard for quantifying the results.

Z and Z_{out} exhibited comparable, marked separation performance (Fig. S1). In particular, the maximum CO_2/CH_4 separation factors (SFs) for Z and Z_{out} were ca. 529 ± 45 (Fig. S1a) and 488 ± 110 (Fig. S1b), respectively, at 30 °C. The corresponding CO_2 permeances at 30 °C were the highest among the measured temperatures; approximately (1.53 ± 0.20) × 10^{-6} mol·m^{-2}·s^{-1}·Pa^{-1} for Z and (1.34 ± 0.29) × 10^{-6} mol·m^{-2}·s^{-1}·Pa^{-1} for Z_{out}. Notably, as the temperature increased, the CO_2 permeance decreased monotonically, whereas the CH_4 permeance remained relatively constant. Consequently, the CO_2/CH_4 SFs of both Z and Z_{out} decreased monotonically with increasing temperature (Fig. S1). Considering that the CO_2/CH_4 separation performance is a sensitive function of 8-membered-ring zeolite membranes,[5] the membrane qualities of both Z and Z_{out} synthesized in this study were comparable to each other and equivalent to those of our previously reported DDR@CHA hybrid membrane.[2]

S2.2. Tol demethylation reaction during long-term stability test of the G-based packed-bed reactor for MCH dehydrogenation

As shown in Fig. 4a, although the Tol yields at 300 °C were as high as ca. 99.6%, the molar flow rates of benzene and methane increased and the Tol yields decreased simultaneously right after increasing the reaction temperature due to the Tol demethylation reaction. Subsequently, the molar flow rates of benzene and methane gradually decreased, and finally became asymptotic. This can be ascribed to the hindrance of Tol demethylation by mild catalyst deactivation.[6]

Table S1. Numerical values of permeances and separation factors through Z and the corresponding molar fluxes and H_2 purities measured at H_2/Tol molar ratio of 3:1 in the mixed feed shown in Fig. 2a1-a2. Separation performance tests were performed with 50 mL·min^{-1} of Ar sweep gas at different temperatures at 1 bar on the feed side. For the H_2 purity, the Ar sweep gas was excluded from the calculation.

**Temperature** (℃)	**Permeance**		**Separation Factor**	**Molar Flux**		**Purity**
	H_2 (mol·m^{-2}·s^{-1}·Pa^{-1}) × 10^8	Tol (mol·m^{-2}·s^{-1}·Pa^{-1}) × 10^{10}	H_2/Tol	H_2 (mol·m^{-2}·s^{-1}) × 10^3	Tol (mol·m^{-2}·s^{-1}) × 10^6	H_2 (%)
190	10.14 ± 0.21	3.53 ± 0.40	353 ± 48	10.19 ± 0.06	9.65 ± 1.26	99.91 ± 0.01
220	10.56 ± 0.53	3.21 ± 0.43	406 ± 59	10.62 ± 0.35	8.78 ± 1.01	99.92 ± 0.01
250	9.84 ± 0.15	3.06 ± 0.18	403 ± 17	9.89 ± 0.17	8.26 ± 0.44	99.92 ± 0.003
275	9.81 ± 0.17	3.13 ± 0.46	377 ± 41	9.36 ± 0.26	8.31 ± 1.23	99.91 ± 0.01
300	9.65 ± 0.33	3.50 ± 0.23	375 ± 32	9.09 ± 0.23	9.56 ± 0.90	99.90 ± 0.01

Table S2. Numerical values of permeances and separation factors through Z and the corresponding molar fluxes and H_2 purities measured at H_2/MCH molar ratio of 3:1 in the mixed feed shown in Fig. 2b1-b2. Separation performance tests were performed with 50 mL·min^{-1} of Ar sweep gas at different temperatures at 1 bar on the feed side. For the H_2 purity, the Ar sweep gas was excluded from the calculation.

**Temperature** (°C)	**Permeance**		**Separation Factor**	**Molar Flux**		**Purity**
	H_2 (mol·m^{-2}·s^{-1}·Pa^{-1}) × 10^8	MCH (mol·m^{-2}·s^{-1}·Pa^{-1}) × 10^{10}	H_2/MCH	H_2 (mol·m^{-2}·s^{-1}) × 10^3	MCH (mol·m^{-2}·s^{-1}) × 10^6	H_2 (%)
190	9.51 ± 1.14	2.62 ± 0.13	441 ± 48	9.49 ± 1.05	6.96 ± 0.40	99.93 ± 0.01
220	9.18 ± 0.75	2.53 ± 0.18	437 ± 18	8.99 ± 0.65	6.81 ± 0.57	99.92 ± 0.001
250	10.48 ± 0.77	3.11 ± 0.34	470 ± 76	9.56 ± 0.18	7.63 ± 0.86	99.92 ± 0.01
275	9.43 ± 0.28	2.37 ± 0.18	467 ± 18	9.06 ± 0.40	6.43 ± 0.34	99.93 ± 0.001
300	9.44 ± 0.19	2.26 ± 0.46	488 ± 79	9.25 ± 0.07	5.56 ± 1.32	99.94 ± 0.01

Table S3. Numerical values of permeances and separation factors through Z_{out} and the corresponding molar fluxes and H_2 purities measured at H_2/Tol molar ratio of 3:1 in the mixed feed shown in Fig. S5a1-a2. Separation performance tests were performed with 50 mL·min^{-1} of Ar sweep gas at different temperatures at 1 bar on the feed side. For the H_2 purity, the Ar sweep gas was excluded from the calculation.

**Temperature** (°C)	**Permeance**		**Separation Factor**	**Molar Flux**		**Purity**
	H_2 (mol·m^{-2}·s^{-1}·Pa^{-1}) × 10^8	Tol (mol·m^{-2}·s^{-1}·Pa^{-1}) × 10^{10}	H_2/Tol	H_2 (mol·m^{-2}·s^{-1}) × 10^3	Tol (mol·m^{-2}·s^{-1}) × 10^6	H_2 (%)
190	9.79 ± 0.12	3.08 ± 0.15	342 ± 56	9.11 ± 0.41	9.30 ± 0.59	99.90 ± 0.01
220	9.22 ± 0.27	3.11 ± 0.65	329 ± 6	8.75 ± 0.32	9.39 ± 0.14	99.89 ± 0.004
250	9.09 ± 0.27	2.82 ± 0.23	343 ± 21	8.63 ± 0.07	8.52 ± 0.91	99.90 ± 0.01
275	9.56 ± 0.81	2.82 ± 0.17	351 ± 23	9.08 ± 0.15	8.51 ± 0.14	99.91 ± 0.001
300	9.47 ± 1.47	2.77 ± 0.23	354 ± 23	8.99 ± 0.43	8.37 ± 0.67	99.91 ± 0.01

Table S4. Numerical values of permeances and separation factors through Z_{out} and the corresponding molar fluxes and H_2 purities measured at H_2/MCH molar ratio of 3:1 in the mixed feed shown in Fig. S5b1-b2. Separation performance tests were performed with 50 mL·min^{-1} of Ar sweep gas at different temperatures at 1 bar on the feed side. For the H_2 purity, the Ar sweep gas was excluded from the calculation.

**Temperature** (°C)	**Permeance**		**Separation Factor**	**Molar Flux**		**Purity**
	H_2 (mol·m^{-2}·s^{-1}·Pa^{-1}) × 10^8	MCH (mol·m^{-2}·s^{-1}·Pa^{-1}) × 10^{10}	H_2/MCH	H_2 (mol·m^{-2}·s^{-1}) × 10^3	MCH (mol·m^{-2}·s^{-1}) × 10^6	H_2 (%)
190	9.00 ± 0.40	2.03 ± 0.08	352 ± 18	7.26 ± 0.25	4.92 ± 0.26	99.93 ± 0.002
220	7.22 ± 0.15	2.01 ± 0.23	336 ± 37	5.82 ± 0.36	4.89 ± 0.43	99.92 ± 0.01
250	8.05 ± 0.61	2.01 ± 0.17	355 ± 13	6.49 ± 0.86	4.88 ± 0.65	99.92 ± 0.02
275	8.30 ± 0.28	2.03 ± 0.35	333 ± 10	6.70 ± 0.88	4.92 ± 0.84	99.93 ± 0.004
300	7.71 ± 0.34	1.82 ± 0.11	346 ± 16	6.22 ± 0.17	4.43 ± 0.92	99.93 ± 0.01

Table S5. Numerical values of permeances and separation factors through Z and the corresponding molar fluxes and H_2 purities measured at H_2/Tol molar ratio of 3:1 in the mixed feed in vacuum mode shown in Fig. 5a1-a2.

**Temperature** (°C)	**Permeance**		**Separation Factor**	**Molar Flux**		**Purity**
	H_2 ($mol \cdot m^{-2} \cdot s^{-1} \cdot Pa^{-1}$) $\times 10^8$	Tol ($mol \cdot m^{-2} \cdot s^{-1} \cdot Pa^{-1}$) $\times 10^{10}$	H_2/Tol	H_2 ($mol \cdot m^{-2} \cdot s^{-1}$) $\times 10^3$	Tol ($mol \cdot m^{-2} \cdot s^{-1}$) $\times 10^6$	H_2 (%)
190	15.80 ± 0.27	1.29 ± 0.15	1105 ± 124	11.94 ± 0.28	3.31 ± 0.49	99.97 ± 0.01
220	16.56 ± 0.91	1.47 ± 0.07	992 ± 84	11.99 ± 0.56	4.22 ± 0.05	99.96 ± 0.002
250	16.63 ± 2.49	1.96 ± 0.17	740 ± 184	12.05 ± 1.61	5.27 ± 0.25	99.96 ± 0.01

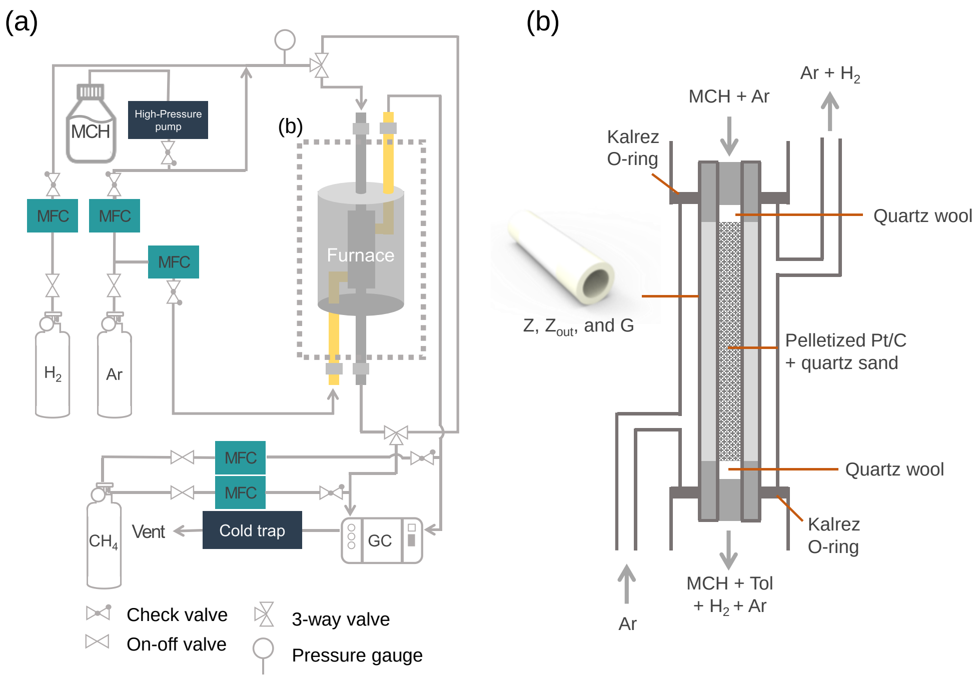

Scheme S1. Schematics of (a) an experimental setup for the MCH dehydrogenation reaction and (b) the MR cell inside the furnace, indicated by a gray dotted box in (a).

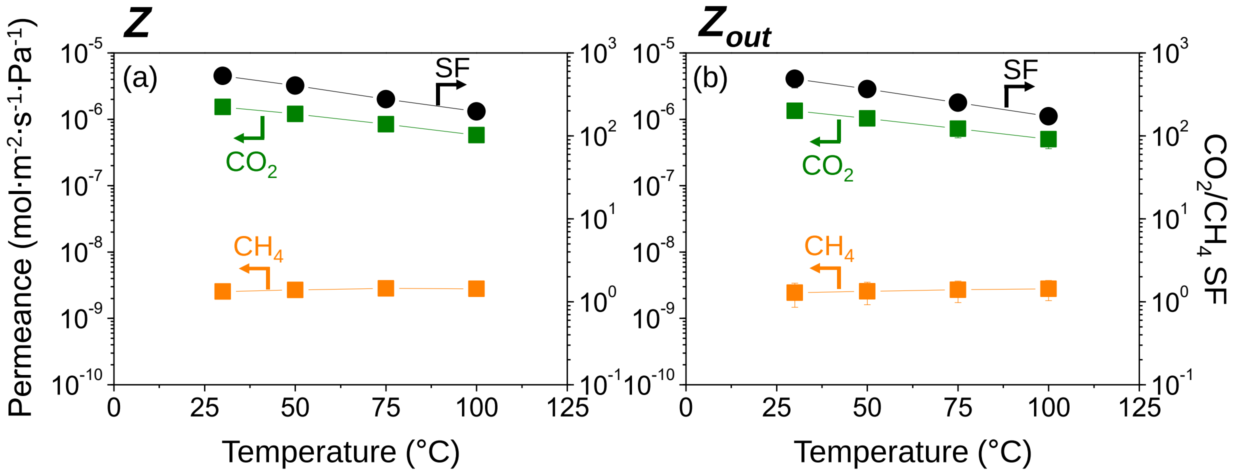

Fig. S1. CO_2 and CH_4 permeances and CO_2/CH_4 SFs of (a) Z and (b) Z_{out} measured for an equimolar CO_2/CH_4 binary mixture at a flow rate of 1000 $mL·min^{-1}$ in vacuum mode. Measurements were carried out at 30, 50, 75, and 100 °C. Data shown here are averaged results of three independent samples and error bars (representing the corresponding standard deviations) are included for all data points. For clarity, the error bars that are embedded in the symbols (so not conspicuous) are owing to the low standard deviation values.

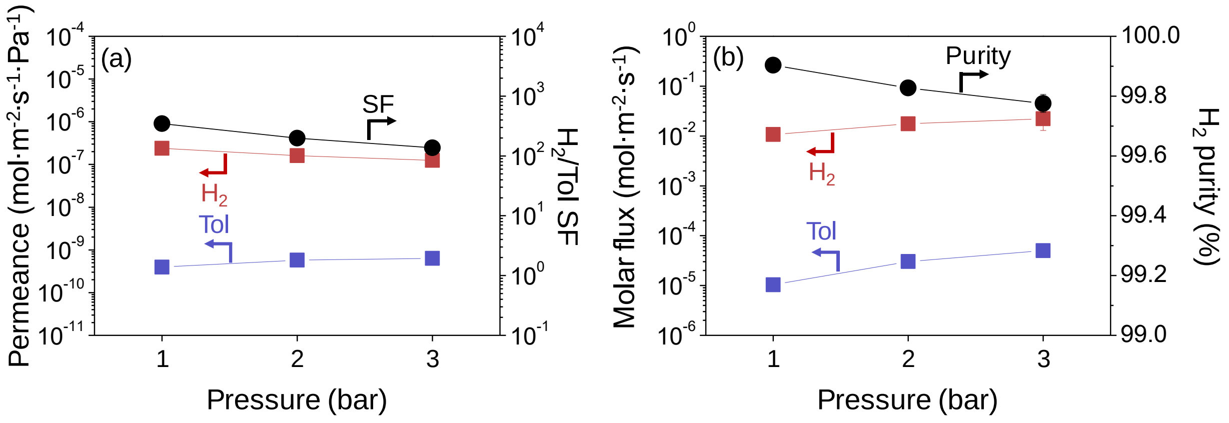

Fig. S2. (a) H_2 and Tol permeances and H_2/Tol SFs of Z and (b) the corresponding H_2 and Tol molar fluxes and H_2 purities. Separation performance tests were performed with 50 mL·min^{-1} of Ar sweep gas at 1, 2, and 3 bar on the feed side at 220 °C. For the H_2 purity in (b), the Ar sweep gas was excluded from the calculation. Data shown here are averaged results of three independent samples and error bars (representing the corresponding standard deviations) are included for all data points. For clarity, the error bars that are embedded in the symbols (so not conspicuous) are owing to the low standard deviation values.

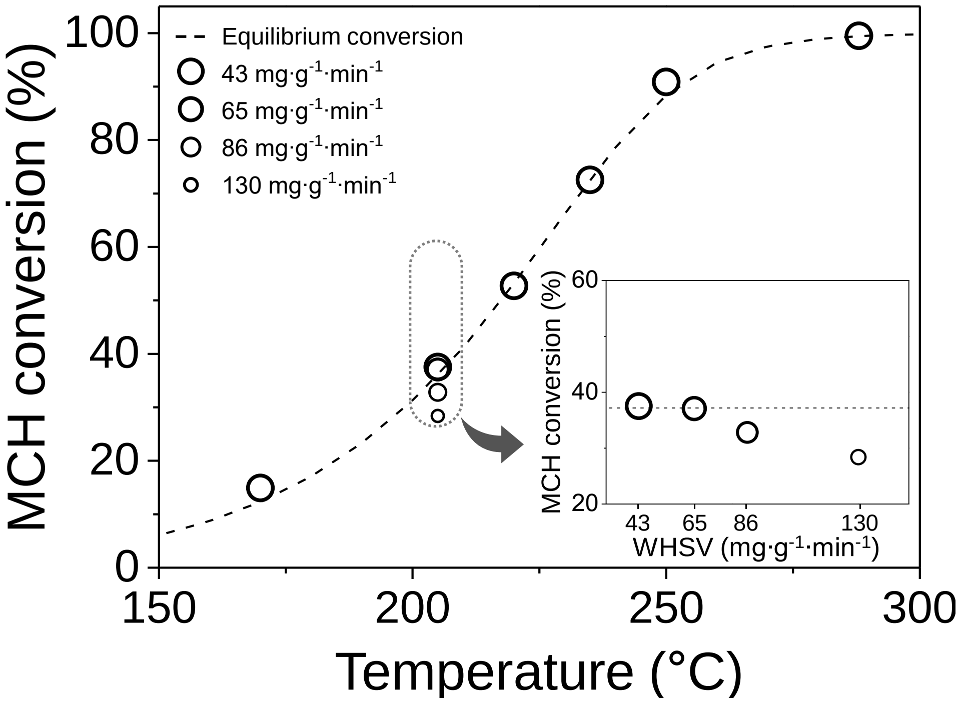

Fig. S3. MCH conversions with a quartz reactor (Q) during the MCH dehydrogenation reaction. The MCH conversion curve at equilibrium was calculated using Equation (S5) and is marked by a gray-dashed curve. The MCH dehydrogenation reactions were conducted at WHSVs of 43, 65, 86, and 130 mg·g^{-1}·min^{-1} at 205 °C and temperatures of ca. 170, 205, 220, 235, 250, and 288 °C at a WHSV of 43 mg·g^{-1}·min^{-1}. Reaction conditions: P_{Total} = 1 bar and flow rate of carrier gas (Ar) = 10 mL·min^{-1}.

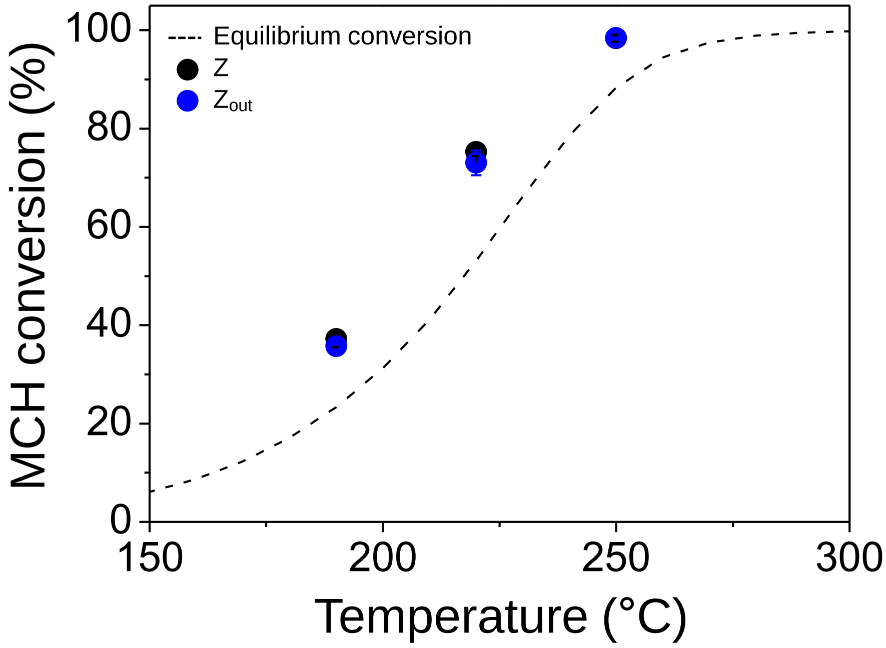

Fig. S4. MCH conversions with Z- and Z_{out}-based MRs during the MCH dehydrogenation reactions. The MCH conversion curve at equilibrium was obtained using Equation (S5) and is marked by a gray-dashed curve. The MCH dehydrogenation reactions were conducted at 190, 220, and 250 °C. Reaction condition: P_{Total} = 1 bar, WHSV = 7.7 mg·g^{-1}·min^{-1}, flow rate of carrier gas (Ar) = 10 mL·min^{-1}, and flow rate of sweep gas (Ar) = 50 mL·min^{-1}. Data shown here are averaged results of three independent samples and error bars (representing the corresponding standard deviations) are included for all data points. For clarity, the error bars that are embedded in the symbols (so not conspicuous) are owing to the low standard deviation values.

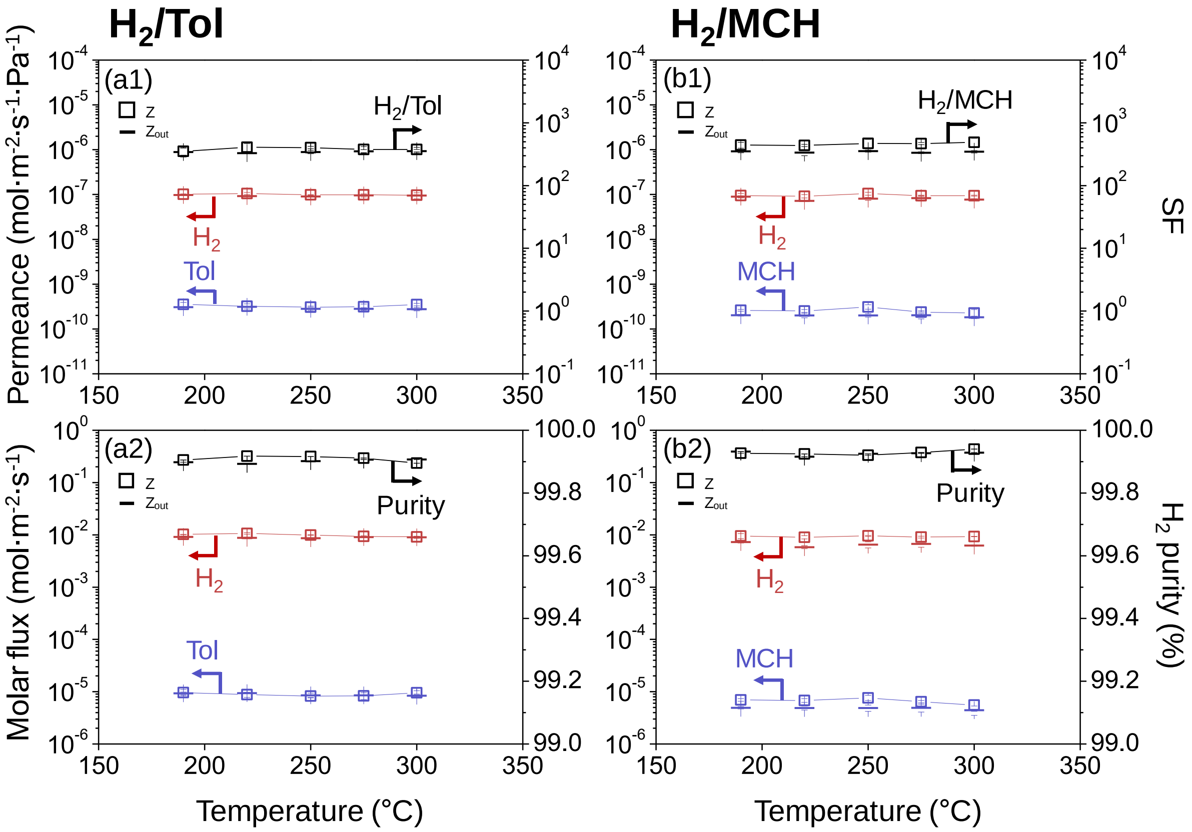

Fig. S5. (a1)-(b1) Permeances and SFs through Z and Z_{out} and (a2)-(b2) the corresponding molar fluxes and H_2 purities measured at (a1)-(a2) H_2/Tol and (b1)-(b2) H_2/MCH molar ratios of 3:1 in the mixed feed. Separation performance tests were performed with 50 mL·min^{-1} of Ar sweep gas at different temperatures (i.e., 190, 220, 250, 275, and 300 °C) at 1 bar on the feed side. For the H_2 purity in (a2)-(b2), the Ar sweep gas was excluded from the calculation. Data shown here are averaged results of three independent samples and error bars (representing the corresponding standard deviations) are included for all data points. For clarity, the error bars that are embedded in the symbols (so not conspicuous) are owing to the low standard deviation values.

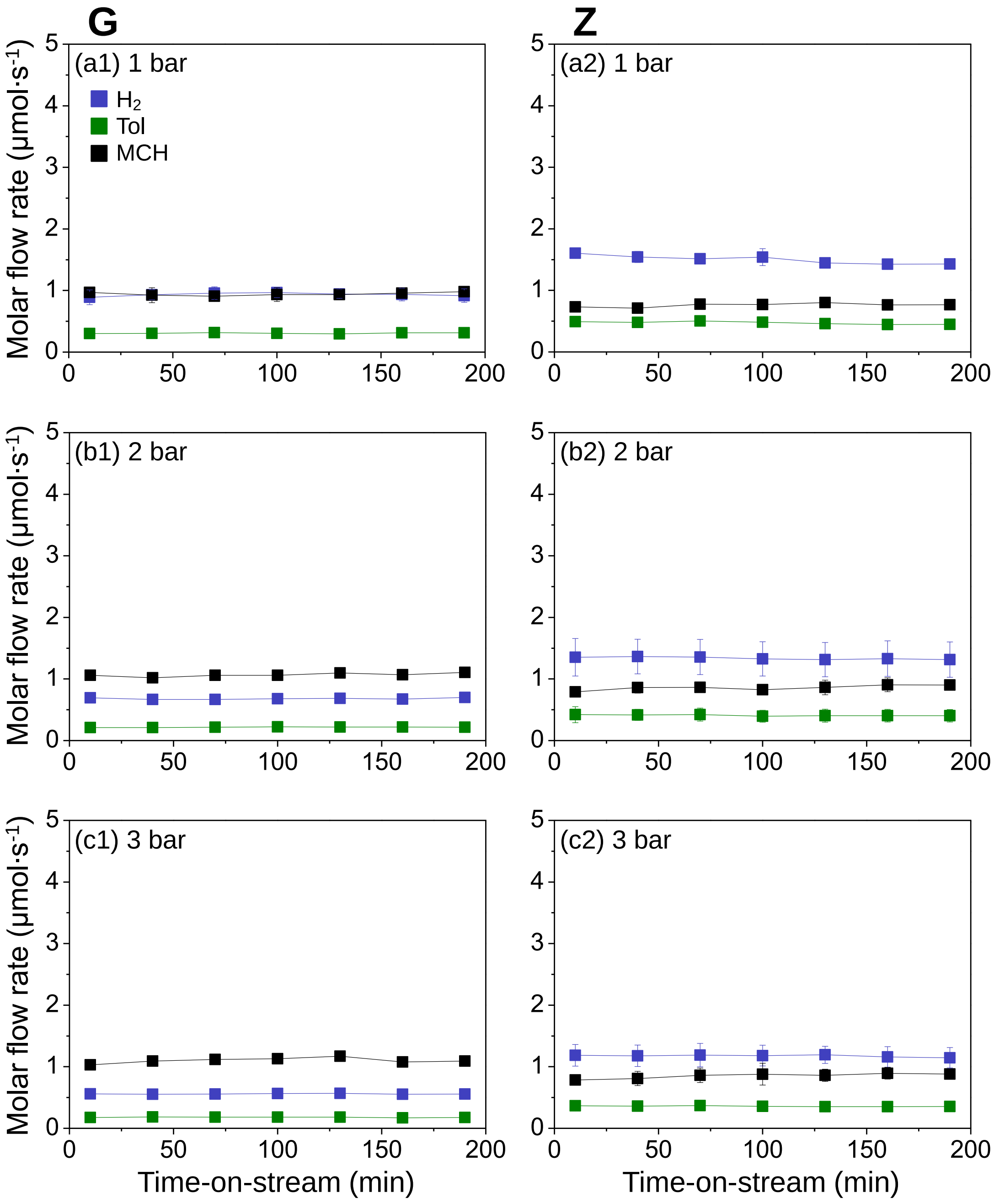

Fig. S6. Molar flow rates of H_2, Tol, and MCH as a function of time-on-stream in (a1)-(c1) G-based packed-bed reactor and (a2)-(c2) Z-based MR for the MCH dehydrogenation reactions at 190 °C: (a1)-(a2) 1, (b1)-(b2) 2, and (c1)-(c2) 3 bar. Reaction conditions: Temp. = 190 °C, WHSV = 7.7 mg·g^{-1}·min^{-1}, flow rate of carrier gas (Ar) = 10 mL·min^{-1}, and flow rate of sweep gas (Ar) = 50 mL·min^{-1}. Data shown here are averaged results of three independent samples and error bars (representing the corresponding standard deviations) are included for all data points. For clarity, the error bars that are embedded in the symbols (so not conspicuous) are owing to the low standard deviation values.

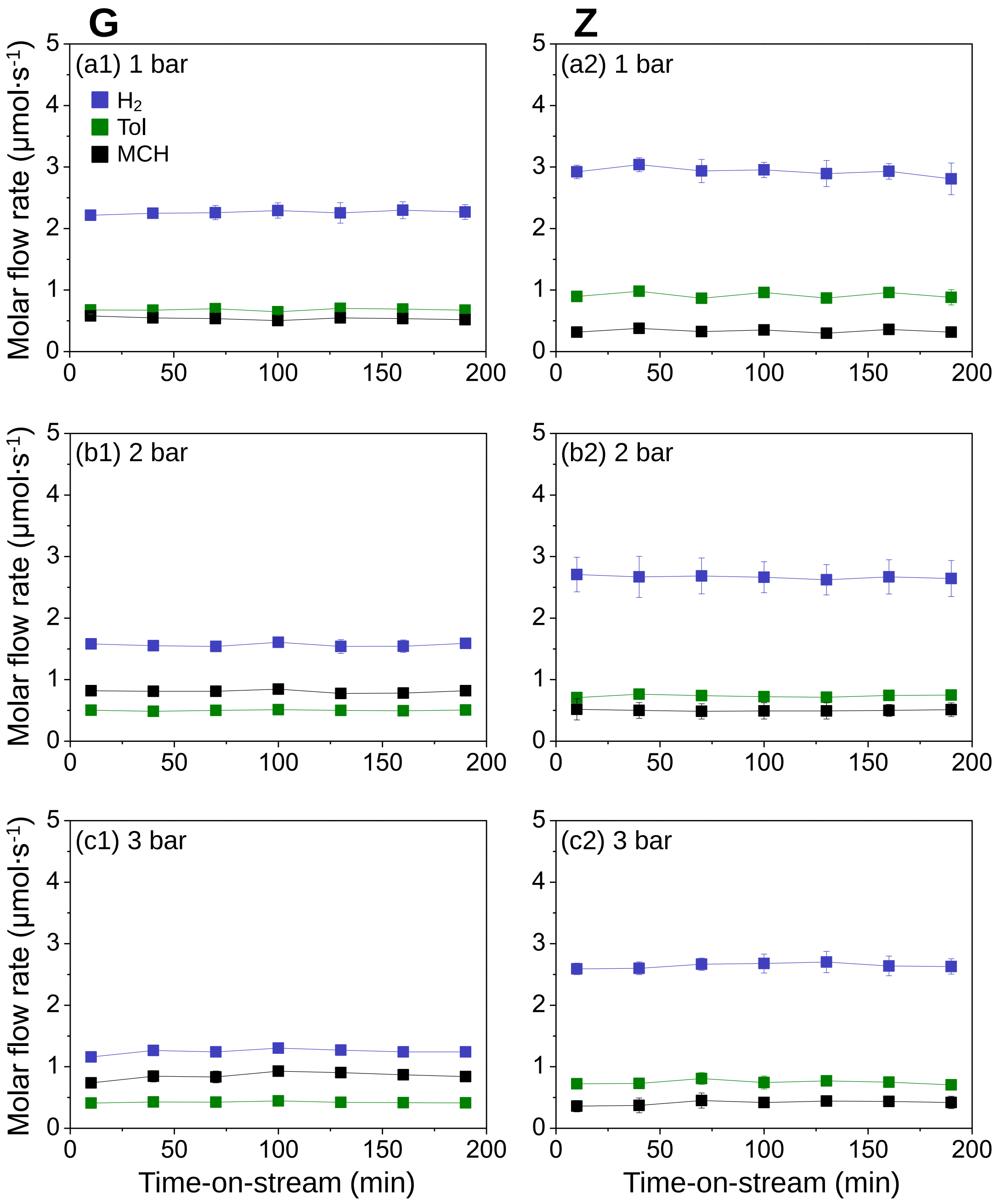

Fig. S7. Molar flow rates of H_2, Tol, and MCH as a function of time-on-stream in (a1)-(c1) G-based packed-bed reactor and (a2)-(c2) Z-based MR for the MCH dehydrogenation reactions at 220 °C: (a1)-(a2) 1, (b1)-(b2) 2, and (c1)-(c2) 3 bar. Reaction conditions: Temp. = 220 °C, WHSV = 7.7 mg·g^{-1}·min^{-1}, flow rate of carrier gas (Ar) = 10 mL·min^{-1}, and flow rate of sweep gas (Ar) = 50 mL·min^{-1}. Data shown here are averaged results of three independent samples and error bars (representing the corresponding standard deviations) are included for all data points. For clarity, the error bars that are embedded in the symbols (so not conspicuous) are owing to the low standard deviation values.

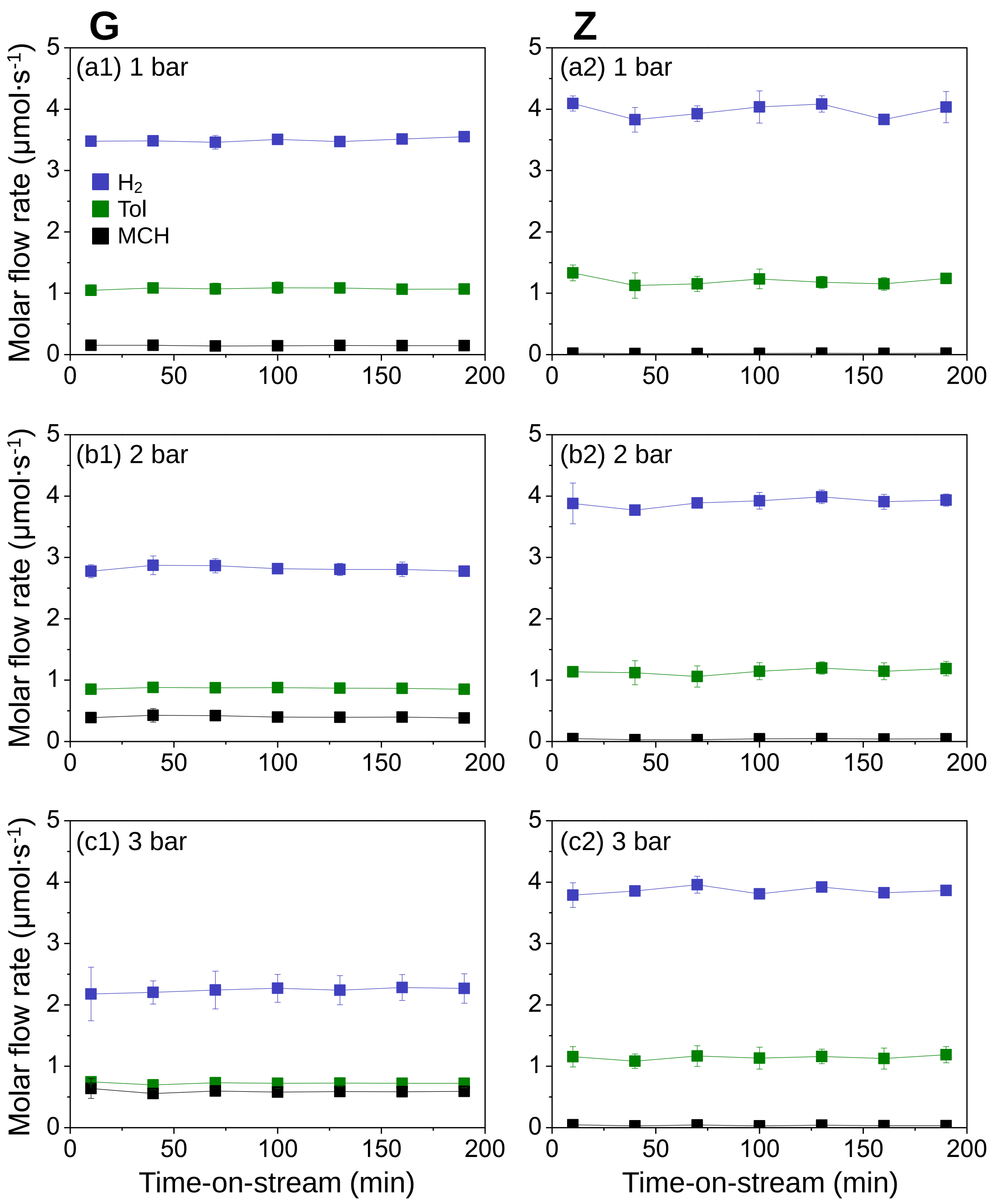

Fig. S8. Molar flow rates of H_2, Tol, and MCH as a function of time-on-stream in (a1)-(c1) G-based packed-bed reactor and (a2)-(c2) Z-based MR for the MCH dehydrogenation reactions at 250 °C: (a1)-(a2) 1, (b1)-(b2) 2, and (c1)-(c2) 3 bar. Reaction conditions: Temp. = 250 °C, WHSV = 7.7 mg·g^{-1}·min^{-1}, flow rate of carrier gas (Ar) = 10 mL·min^{-1}, and flow rate of sweep gas (Ar) = 50 mL·min^{-1}. Data shown here are averaged results of three independent samples and error bars (representing the corresponding standard deviations) are included for all data points. For clarity, the error bars that are embedded in the symbols (so not conspicuous) are owing to the low standard deviation values.

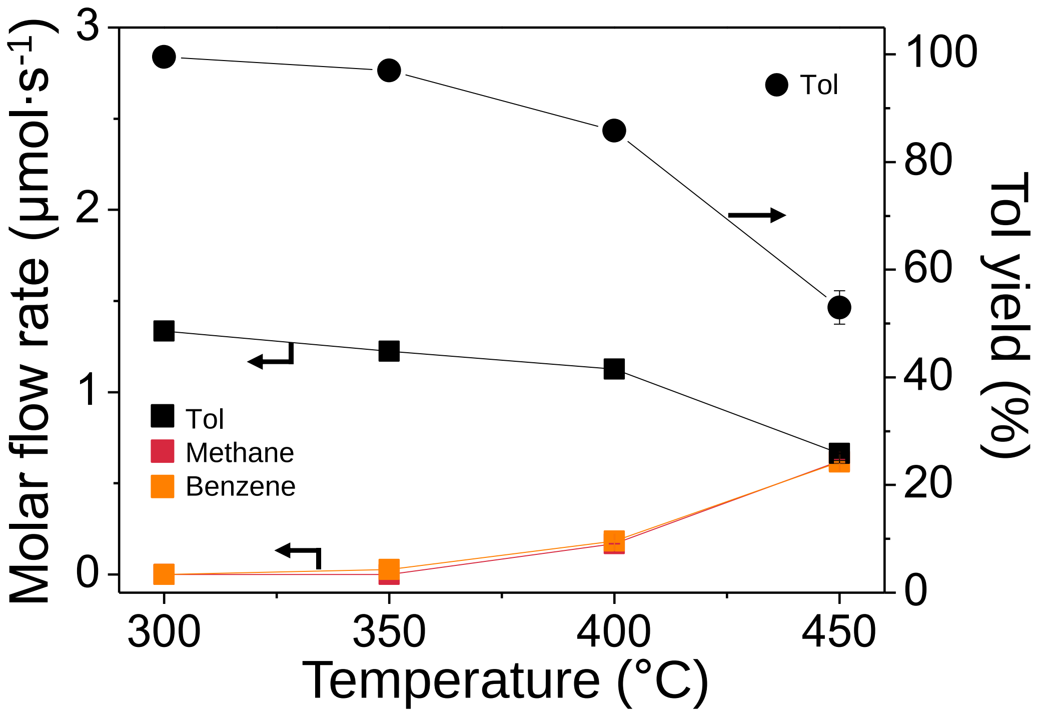

Fig. S9. Molar flow rates of hydrocarbon products (Tol, methane, and benzene) and Tol yield with G-based packed-bed reactor as a function of the reaction temperature in the MCH dehydrogenation reaction. The results were obtained after 2-h time elapse at each temperature. Data shown here are averaged results of three independent samples and error bars (representing the corresponding standard deviations) are included for all data points. For clarity, the error bars that are embedded in the symbols (so not conspicuous) are owing to the low standard deviation values.

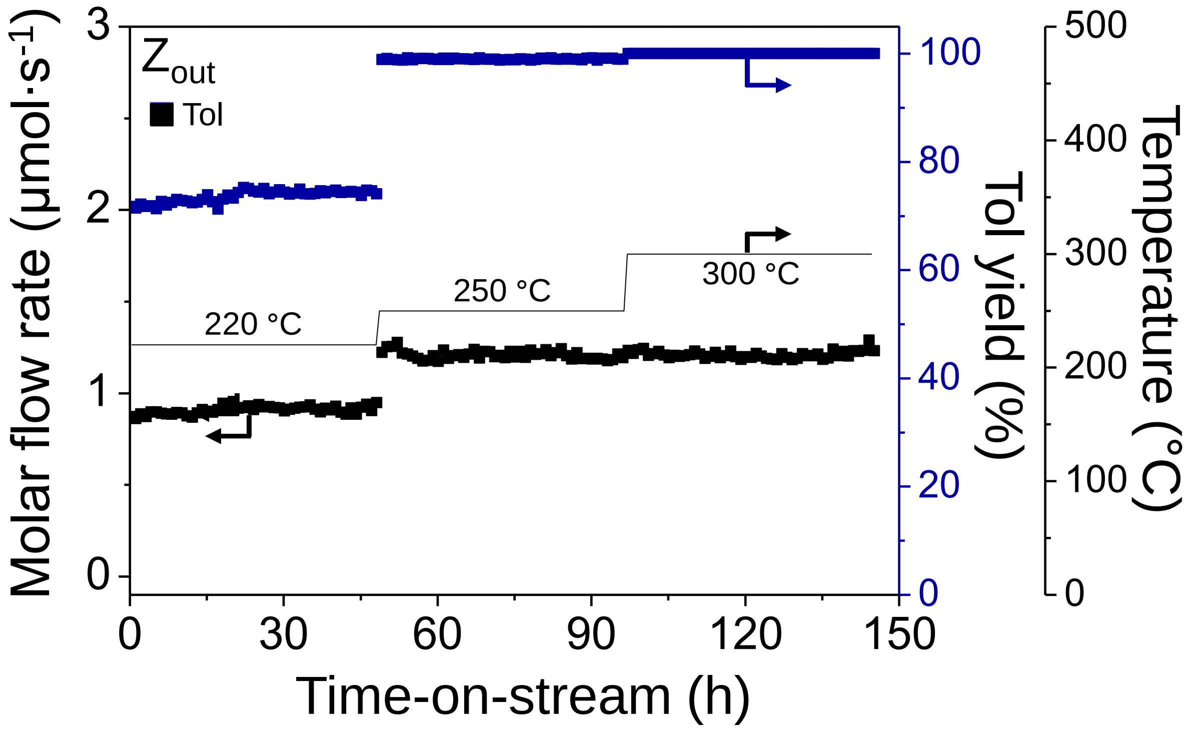

Fig. S10. Molar flow rates of hydrocarbon products and Tol yields with the Z_{out}-based MR as a function of time-on-stream during MCH dehydrogenation reactions. The reactions at each temperature (220, 250, or 300 °C) were conducted for 48 h. Only Tol was detected as a hydrocarbon product. Reaction condition: P_{Total} = 1 bar, WHSV = 7.7 mg·g^{-1}·min^{-1}, flow rate of carrier gas (Ar) = 10 mL·min^{-1}, and flow rate of sweep gas (Ar) = 50 mL·min^{-1}.

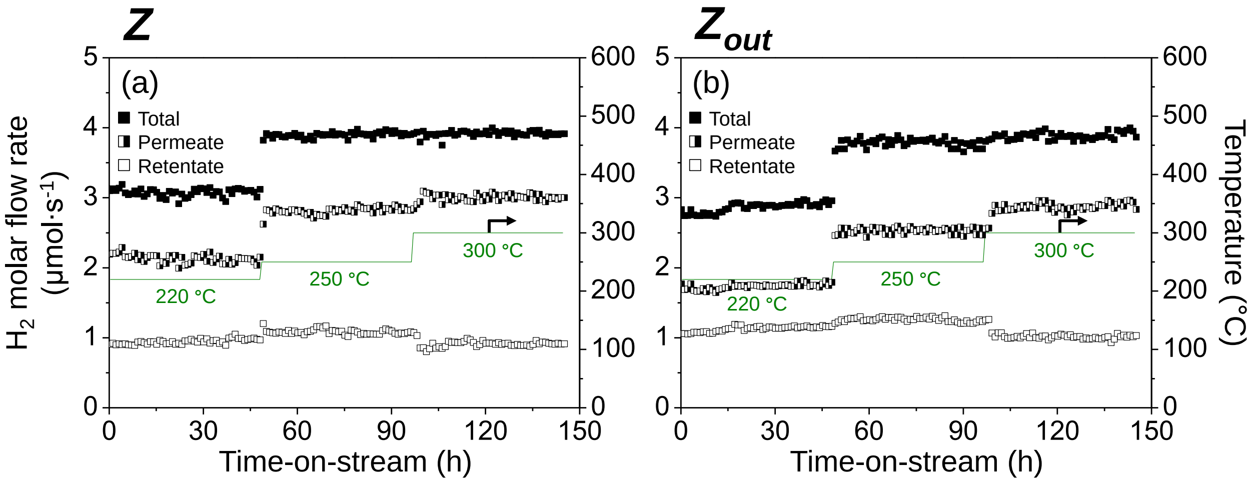

Fig. S11. H_2 molar flow rate with (a) Z- and (b) Z_{out}-based MRs as a function of time-on-stream during the MCH dehydrogenation reaction. The total H_2 molar flow rates (filled symbols) were obtained by combining the molar flow rates of H_2 on the permeate (half-filled symbols) and retentate (empty symbols) sides.

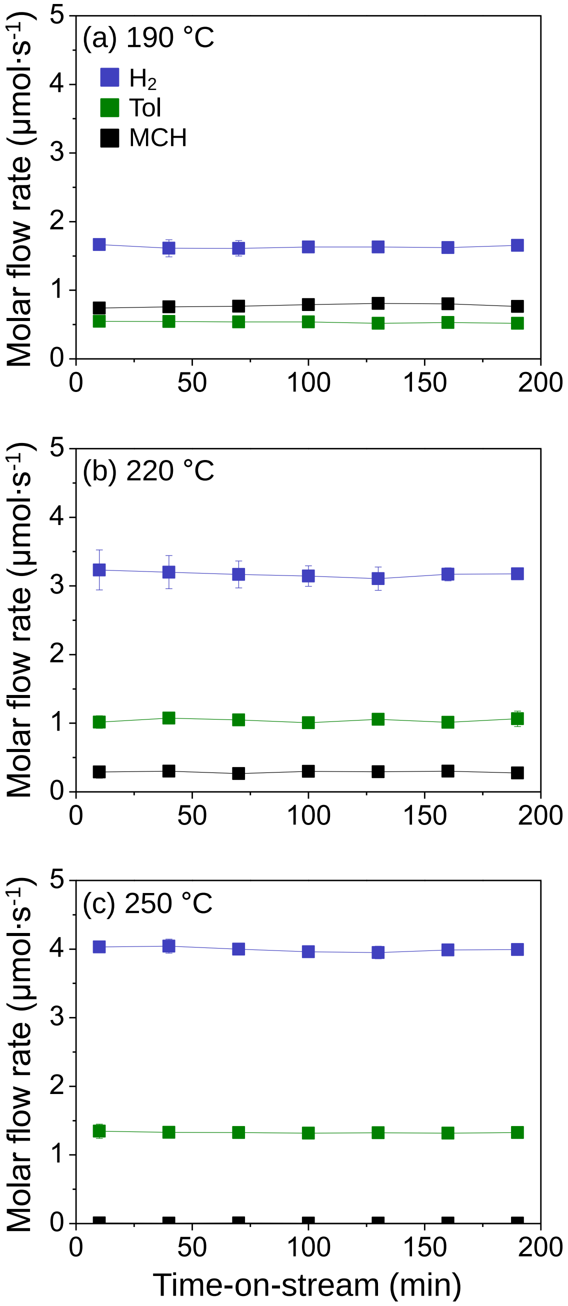

Fig. S12. Molar flow rates of H_2, Tol, and MCH as a function of time-on-stream in the Z-based MR for the MCH dehydrogenation reactions at (a) 190, (b) 220, and (c) 250 °C in vacuum mode; the pressure on the permeate side was maintained at ca. 0.03 bar. Reaction conditions: WHSV = 7.7 mg·g^{-1}·min^{-1}, flow rate of carrier gas (Ar) = 10 mL·min^{-1}, and flow rate of sweep gas (Ar) = 50 mL·min^{-1}. Data shown here are averaged results of three independent samples and error bars (representing the corresponding standard deviations) are included for all data points. For clarity, the error bars that are embedded in the symbols (so not conspicuous) are owing to the low standard deviation values.

Reference

[1] Y. Jeong, S. Hong, E. Jang, E. Kim, H. Baik, N. Choi, A. C. K. Yip, J. Choi, *Angew. Chem. Int. Edit.* **2019**, *58*, 18654-18662.

[2] Y. Jeong, S. Kim, M. Lee, S. Hong, M. G. Jang, N. Choi, K. S. Hwang, H. Baik, J. K. Kim, A. C. K. Yip, J. Choi, *ACS Appl. Mater. Interfaces* **2022**, *14*, 2893-2907.

[3] M. J. den Exter, J. C. Jansen, H. van Bekkum, *Stud. Surf. Sci. Catal* **1994**, *84*, 1159-1166.

[4] T. Schildhauer, E. Newson, S. Muller, *J. Catal.* **2001**, *198*, 355-358.

[5] L. Yu, M. S. Nobandegani, J. Hedlund, *J. Membr. Sci.* **2022**, *641*, 119888.

[6] F. Alhumaidan, D. Tsakiris, D. Cresswell, A. Garforth, *Int. J. Hydrogen Energy* **2013**, *38*, 14010-14026.
